# In Vivo Confocal Microscopy in Different Types of Dry Eye and Meibomian Gland Dysfunction

**DOI:** 10.3390/jcm11092349

**Published:** 2022-04-22

**Authors:** Ralene Sim, Kenneth Yong, Yu-Chi Liu, Louis Tong

**Affiliations:** 1 Singapore Eye Research Institute, Singapore National Eye Centre, Singapore 168751, Singapore; ralene.sim@mohh.com.sg (R.S.); liuchiy@gmail.com (Y.-C.L.); 2 Yong Loo Lin School of Medicine, National University of Singapore, Singapore 119228, Singapore; e0345868@u.nus.edu; 3 Ophthalmology & Visual Sciences Academic Clinical Program (Eye ACP), Duke-NUS Medical School, Singapore 169857, Singapore

**Keywords:** diagnostic device, dry eye, in vivo confocal microscopy (IVCM), inflammation, ocular surface, tear disorder, review

## Abstract

In vivo confocal microscopy (IVCM) imaging is increasingly popular in ocular surface disease diagnosis and management. We conducted a systematic review to update the use of IVCM in the diagnosis and treatment of dry eye and meibomian gland dysfunction (MGD). A literature review was conducted on IVCM studies in MGD, dry eye disease, systemic disease causing dry eye, dry eye in glaucoma patients, contact lens-associated ocular conditions, graft-versus-host disease, and Sjogren’s syndrome-related dry eye. The articles were identified through PubMed and a total number of 63 eligible publications were analyzed in detail. All primary research studies on confocal microscopy on dry eye and related conditions from 2017 onwards were included. The reports were reviewed for their contribution to the existing literature as well as potential biases and drawbacks. Despite limitations such as small field of view, lack of population-based norms, and lack of standardization of image acquisition, interpretation, and quantification, IVCM is useful as a complementary technique for clinical diagnosis in various ocular surface disorders related to dry eye. With advances in hardware and software in the near future, it has the potential for further practical impact.

## 1. Introduction

There are many common ocular surface disorders (OSD), such as Dry Eye Disease (DED), blepharitis, and meibomian gland dysfunction (MGD), whose management requires visualization of certain ocular surface structures via slit-lamp biomicroscopy. In vivo confocal microscopy (IVCM), a more recent imaging technique has been evaluated in clinics for similar visualization [1].

The ocular surface consists of the conjunctiva, cornea, and the ocular mucosal adnexa (eyelid margins, eyelid glands, and lacrimal apparatus). The cornea, though uniquely suitable for IVCM due to its transparency, is not the only structure that can be visualized with this technique. It consists of the epithelium, basement membrane, Bowman’s layer, stroma, pre-Descemet, Descemet membrane, and endothelium [2]. The stroma contains keratocytes, dendritic cells (DCs), and nerve bundles that give rise to multiple branches which penetrate the epithelium [1]. Many of these corneal structures are not visible at the cellular level by conventional slit-lamp biomicroscopy but are clearly visible on IVCM. Unlike conventional light microscopy, IVCM directs light with a 670-nm wavelength laser [3] to pass to the desired focal spot using a pinhole aperture, which overcomes the problem of light scattering and provides clearer images at the cellular level. The resolution and magnification of IVCM (800-fold) are also much better than that of slit-lamp microscopy (40 fold), thus allowing improved and even cellular resolution of the ocular surface [4]. The resolution is also superior, with a lateral resolution of 1–2 μm and axial resolution of 5–10 μm [5]. Previously, Cruzat et al. [6] wrote an extensive review in 2017 on the pathological changes of corneal nerves in various ocular surface diseases. Other recent reviews [7,8] published in 2020 have not delved in-depth into various clinical applications of IVCM nor covered the spectrum of non-infective ocular surface diseases, and hence, there is a need for an update. In this article, we aim to review the literature on the clinical use of IVCM, focusing on studies published after 2017.

## 2. Materials and Methods

### 2.1. Study Objective and Definition of Reference Standard

**Figure 1:**
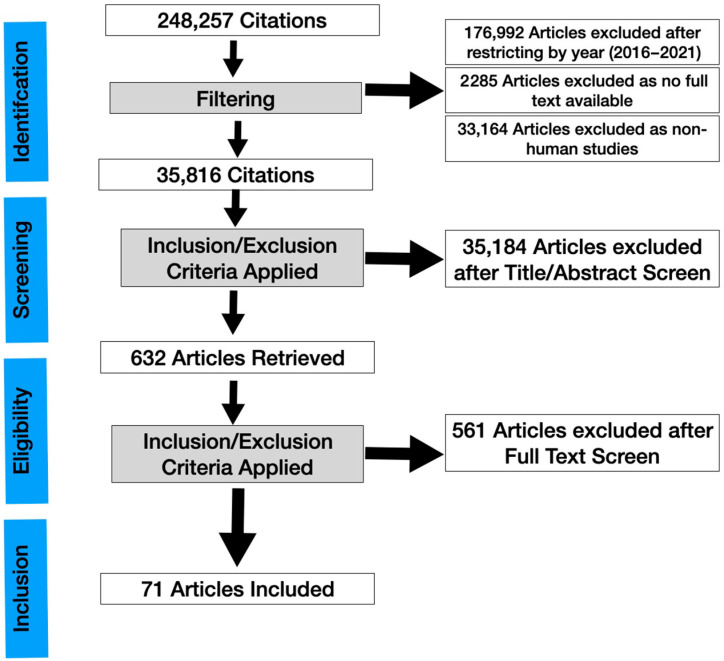
PRISMA flowchart of a systematic review of in vivo confocal microscopy in different types of dry eye and meibomian gland dysfunction.

A systematic review was conducted (Figure 1) and the results were reported according to the Preferred Reporting Items for Systematic Reviews and Meta-Analysis (PRISMA) statement. The main objective of this systematic review and meta-analysis is to evaluate the clinical applications of IVCM in dry eye, focusing on new observations. Because this study did not directly involve patients, an ethics committee approval was not required.

### 2.2. Literature Search Strategy

We determined the criteria and search strategy before the start of the study. An entrez PubMed literature search of the PubMed database using the terms and a detailed combination of keywords to capture ocular surface disease and dry eye was performed from inception until 9 November 2021. The search terms included the following keywords and keyword combinations (((“in vivo” OR in vivo OR in-vivo) AND confocal) OR IVCM) AND keywords to identify a set of ocular surface articles (Please refer to Supplementary File S1).

### 2.3. Eligibility Criteria

Two reviewers (R.S. and K.Y.) screened all retrieved articles by title and abstract initially. Only original research articles written in English were included and not analysis reviews, editorials, opinions, single case reports, and ex-vivo studies. The articles found were curated manually for their relevance to the ocular surface. The following conditions were not considered to be ocular surface disease: infectious keratitis, bullous keratopathy, ocular cystinosis, and Iridocorneal Endothelial Syndrome (ICE) syndrome.

The full text of the remaining studies was curated. Additionally, the reference lists of the remaining studies were checked to identify further relevant articles that may have been overlooked during the initial process. Hard copies of all of the eligible articles were obtained and fully read. 

We excluded articles where IVCM findings were not mentioned in the results of the full-text article. Studies where recovery of the full text was not possible, even after searching the available medical databases and contacting the corresponding authors, were excluded. Disagreements were settled through discussion with a third reviewer (L.T.).

### 2.4. Data Extraction and Quality Evaluation of the Studies

The initial database search with the above keywords identified 248,257 papers. The database search for publication dates from 2016 to 22 November 2021 identified 71,265 studies. 

After excluding articles where full text was not available (2285), 68,980 studies were left. After excluding non-human studies (33,164), 35,816 studies were left. After going through the title and screening through the abstract and applying our inclusion/exclusion criteria (4281 were reviews, 799 were systematic reviews, and 5056 were case reports), 632 studies were left. After full text-retrieval and further curation, 71 studies remained. A risk of bias assessment was then evaluated using the AMSTAR2 tool (Supplementary Table S1) [9] and the methodological quality of eligible articles was assessed using bias using the Revised Cochrane risk-of-bias tool for randomized trials RoB2 tool (Supplementary Table S2) [10]. Supplementary Table S2 presents the risk of bias summary per domain for individual studies and for all 4 included studies. The overall risk of biased judgment has been ascertained to reflect concerns in these studies as they have some concerns in at least one domain.

## 3. Results

### 3.1. Meibomian Gland Dysfunction

Meibomian glands (MG) have been classically described to compose of acini constituted by convoluted borders lined by large cells with fine cellular material within the lumen [11], interstitial space between acini, ductules, and terminal ducts. Abnormal meibum quality and quantity can lead to a decreased or altered tear film lipid layer, tear hyperosmolarity, tear instability, and inflammation, leading to ocular surface damage and DED [12]. Significant fibrosis (demonstrated via loss of MG architecture with extensive fibrotic tissue surrounding MG remnants) has been observed in chronic MG dysfunction [13]. Recent studies on MGD are summarized in Table 1. A decrease in the size of the MG acinar unit was also observed [14]. IVCM has also been used to analyze the palpebral conjunctiva to visualize and quantify the density of immune cells [15]. These cells have been evaluated in different locations: epithelial (EIC), intraglandular (IGIC), stromal (SIC), and periglandular (PGIC) regions. The immune cells in EIC and IGIC were increased in MGD patients with more severe dry eye symptoms, even in those with minimal corneal staining [16]. Basal epithelial cell density was also found to be reduced with greater stromal nerve thickness in the MGD group [17]. Hence IVCM may provide reliable and clinically relevant metrics of inflammation and serve as clinical endpoints in future clinical trials targeting inflammation in MGD.

### 3.2. Dry Eye Disease

Dry eye disease (DED) is defined as a “multifactorial disease of the ocular surface characterized by a loss of homeostasis of the tear film and accompanied by ocular symptoms, in which tear film instability and hyperosmolarity, ocular surface inflammation and damage, and neurosensory abnormalities play etiological roles” [16]. The ocular surface, epithelial sensory receptors, the innervation of the epithelial sensory receptors, secretory centers in the brain, and efferent nerves supplying the meibomian glands, goblet cells, and the main lacrimal gland form a functional unit. Any or all of these structures may be affected in DED [18]. The cornea is the most densely innervated part of the body. The cornea nerves serve the protective blink reflexes, help in tear secretion, and release neurotransmitters necessary for epithelial and stromal support as well as ocular homeostasis. They also serve the nociceptors associated with mechanical stimuli, pain, and cold sensations. Corneal nerves and their morphological changes can be seen under IVCM. With the help of analytic software, the corneal nerve plexuses can be evaluated quantitatively, typically by measuring the nerve fiber density, length, nerve branch density, and tortuosity [19,20]. The IVCM studies related to dry eye are summarized in Table 2.

Corneal dendritic cells (DC) have been shown to be increased in dry eye patients compared to controls [21]. In the pathogenesis of dry eye, DCs play an important role in inducing the activation of T cells [22], thus triggering an inflammatory cascade reaction.

Reduced corneal nerve density and length indicate a greater degree of neural damage induced by ocular pathology [23]. It has been shown that reduced density of corneal nerves results in impairment of protective functions such as tear secretion and blink reflexes. This results in a reduction in the tear quality and even aqueous tear deficiency. A study has suggested that patients with DED (diagnosed using TBUT and Schirmer’s, as well as the presence of symptoms) had decreased corneal nerve density [24].

Nerve tortuosity, defined by the frequency and the amplitude of the variations in the nerve fiber orientation, suggests active regeneration of nerve fibers in damaged nerves [25]. Studies by Liu et al. [26], Tepelus et al. [24], and Baikai et al. [27] have shown that nerve tortuosity is positively correlated with the diagnosis of DED. A greater nerve tortuosity is linked to ocular discomfort, visual function disturbance, and tear film instability [27].

### 3.3. Sjogren’s-Related Dry Eye (SSDE)

Sjogren syndrome (SS) is a systemic autoimmune disease that initially targets the lacrimal and salivary glands primarily, resulting in keratoconjunctivitis sicca (SSDE) and stomatitis sicca (dry mouth). The prevalence of primary SS in the USA approaches 1.3 million, with a range of 0.4–3.1 million [28]. The IVCM studies related to Sjogren’s-related dry eye are summarized in Table 2 as well. Certain IVCM parameters in sub-basal nerves have been reported to be altered in SS. Nerve fiber density is significantly decreased in SS [29,30], and SSDE is associated with greater nerve tortuosity than non-Sjogren’s Syndrome Dry Eye (NSSDE) [29].

Light backscattering (LB) measured in light reflectivity unit (LRU) at the Bowman’s membrane (BM) at 50 μm, 100 μm, and 200 μm deep to the BM has been evaluated in SS using IVCM—this is a measure of corneal inflammation [31]. Higher levels of LB in each corneal layer compared with healthy controls could indicate increased levels of corneal inflammation in SSDE [32].

The corneal epithelium of DED patients shows morphological changes, such as areas of enlarged and irregularly-shaped cells, which can be quantified by IVCM. Compared to controls, the density of superficial epithelial cells was decreased in both the NSDE and SSDE groups [33].

In summary, IVCM represents a reliable technique for examining nerve tortuosity in DED, as well as documenting corneal epithelial changes and immune cell densities in SSDE.

### 3.4. The Use of IVCM to Evaluate the Treatment for Dry Eye

While disease outcomes have typically been measured using symptomatic questionnaires and clinical tools such as Schirmer Test, corneal and conjunctival staining, tear break up time (TBUT), and tear osmolarity, there has been increasing interest to document treatment outcomes with IVCM [34,35,36].

The IVCM studies performed as part of interventional trials in DED are summarized in Table 3. The most common anti-inflammatory treatment for DED is cyclosporin A (CsA), an immunosuppressant and a calcineurin inhibitor. It has been used in several trials since 1986 and continues to be the major anti-inflammatory drug in the treatment of DED. Six months following treatment with topical CsA in SSDE patients [37], symptoms of dry eye documented by the Ocular Surface Disease Index (OSDI) score improved together with a decrease in corneal nerve tortuosity. The same study reported an increase in sub-basal nerve plexus (SNP) density and a decrease in DC density after treatment. Though increased nerve reflectivity was found, the association was not significant. The decrease in DC density was attributed to the decrease in antigen-presenting cells and local inflammation, and the increase in SNP density was due to the normalization of innervation by controlling the inflammatory reaction [37].

Similarly, Laccheri et al. [38] also found a decrease in nerve tortuosity in SSDE after treatment with cyclosporine. They also found a decrease in nerve reflectivity, the number of sub-basal nerves, and an increase in intermediate corneal epithelial cells. The reduction of sub-basal nerves, reflectivity, and nerve tortuosity could be related to decreased nerve growth factor (NGF) post-treatment, though NGF levels were not checked for in this study. This is uniquely expressed in the human limbal basal epithelium, along with its two corresponding receptors: the low-affinity receptor p75NTR and the high-affinity receptor TrkA. The first receptor, when activated, transmits a signal mainly of apoptosis; the latter, when activated, promotes a molecular cascade aimed at the proliferation and cell activation, which replaces apoptotic cells. NGF secretion is stimulated by high levels of IL-1 and TNF during inflammation and hence treatment of DED would reduce inflammation and the level of NGF. The increase in epithelial cell density after using cyclosporine has also been postulated to be due to decreased NGF and reduction of apoptotic signals through the p75NTR [38]. 

The apparent discrepancies in these studies could be due to the difference in severities of the disease or demographic differences in the patients recruited. 

More recently, homologous sera obtained from healthy donors (i.e., allogeneic peripheral blood serum [allo-PBS] and cord blood serum [CBS]) have been proposed as treatment alternatives in patients with severe DED. Both treatments significantly improved corneal SNP parameters, and in particular, nerve density, length, width, and fractal dimension [39]. Giant epithelial cells, beading, and neuromas have also been shown to be decreased. Corneal nerve fractal dimension (CNFrD) is a novel IVCM metric that measures the structural complexity of corneal nerves, and its reduction represents nerve degeneration. It has been demonstrated that the CNFrD value has a diagnostic efficiency comparable with conventional IVCM parameters for identifying diabetic corneal neuropathy [40]. On the other hand, corticosteroids did not alter the quantitative measurements of the corneal SNP even though they decreased corneal DC density [41]. IVCM was used in a clinical trial that evaluated the use of omega-3 fatty acid supplements in DED [42]. These supplementations may have neuroprotective effects on corneal nerves, shown by an increase in corneal total nerve branch density (CTBD) and corneal nerve branch density on the main fiber (CNBD) after 90 days. 

### 3.5. Systemic Disease

Certain systemic diseases associated with DED are shown in Table 4.

Diabetic neuropathy, including diabetic corneal neuropathy, is one of the most common microvascular complications in diabetes [43]. There is increasing evidence to show that impairment of microvascular components is preceded by early neurodegenerative alterations primarily involving small nerve fibers, which can be demonstrated by IVCM [44,45]. Moreover, as small-fiber neuropathic changes can be picked up by IVCM, corneal nerve metrics have been used as surrogate markers for diabetic peripheral neuropathy [46]. Studies have shown that IVCM parameters such as CNFL [47,48], CNBD, CNFD, and CNFrD are reduced in patients with diabetes compared to controls, especially at the inferior whorl site [49,50]. A significant reduction in nerve beading frequency was also reported, which may be due to reduced metabolomic activity in diabetic patients [51]. 

IVCM is useful in analyzing the cornea and MG structures as well as the skin epidermis and dermis in ocular rosacea. It can quantify MG alterations based on meibum reflectivity, inflammation, and fibrosis, which correlated with the number of Demodex mites in both MG and cheek. However, no correlation was found between IVCM scores and both subjective and objective tests of dry eye [52]. 

Graves’ ophthalmopathy (GO) is often associated with DED, the most frequent cause of ocular discomfort in such patients [53]. GO is an autoimmune disease in which autoantibodies to the thyroid-stimulating hormone receptor lead to an inflammatory response in the orbital tissues [54]. Recent studies with IVCM have found changes in corneal nerves and MGs. Abnormal corneal SNP has been reported in active and inactive GO, suggesting nerve degeneration in GO. These central corneal SNP parameters of GO patients were significantly decreased compared with those of controls: corneal nerve fiber density (CNFD), corneal nerve branch density (CNBD), corneal nerve fiber length (CNFL), corneal nerve fiber total branch density (CTBD), corneal nerve fiber area (CNFA), corneal nerve fiber width (CNFW) and corneal nerve fiber fractal dimension (CNFrD). In addition, CNFD and ACNFrD values were significantly lower in the active GO compared with inactive GO patients. However, this study did not adjust for potential differences in DED between GO states [55]. Hence, further studies could further stratify the active TAO further into mild, moderate, and severe states before comparing the difference in nerve parameters.

IVCM also effectively revealed microstructural changes of MGs in eyes with GO and provided strong in vivo evidence for the roles of obstruction and inflammation in the disease process [56]. However, the patients in both groups had differing OSDI scores. Hence, it is unclear if the MG changes are related to concomitant DED in the GO patients or related to an extension of GO orbitopathy.

Previous studies have discovered an association between DED and migraine headaches [57,58]. It has been hypothesized that the trigeminal system plays a critical role in the pathogenesis of ocular symptoms in migraine. The pain or photophobia associated with migraine is believed to arise from the release of vasoactive neuropeptides at the peripheries of the three main branches of the trigeminal nerve, which innervates the dura, cranium, face, and eye. The ophthalmic branch (V1), in particular, also serves as the afferent for ocular discomfort associated with dry eye [59,60]. IVCM can also study the structural changes in nociceptive corneal axons in the SNP, which showed a decrease in corneal nerve fiber length, total branch density, nerve branch density, and fiber area in patients with migraine and photophobia compared to patients with migraine without photophobia. Hence, SNP changes on IVCM may serve as a potential imaging marker for ocular symptoms of chronic migraine [61]. Unfortunately, the study did not include controls without migraine and did not assess the DED parameters in the participants. Hence, it is difficult to conclude whether the presence of DED parameters is indicative of the severity of migraines.

### 3.6. Glaucoma Treatment-Related Dry Eyes

Glaucoma is the leading cause of global irreversible blindness. The number of people with glaucoma worldwide will increase to 111.8 million in 2040, disproportionally affecting people residing in Asia and Africa [62]. Glaucoma is the leading cause of global irreversible blindness. The most common initial treatment for glaucoma is topical medical therapy and about half of glaucoma patients on topical anti-glaucomatous medications have the ocular surface disease [63]. Previous studies have demonstrated that toxic and proinflammatory effects of antiglaucoma ophthalmic solutions are mainly due to preservatives, though prostaglandins by themselves can cause periorbitopathy [64,65].

IVCM studies related to glaucoma are summarized in Table 5. Such imaging is useful in evaluating proinflammatory ocular surface changes induced by anti-glaucoma eye drops. These parameters may be affected: basal epithelial cell density, stromal reflectivity, number of sub-basal nerves, sub-basal nerve tortuosity, sub-basal nerve reflectivity, and endothelial cell density. One study found increased basal epithelial cells density, stromal reflectivity, sub-basal nerve tortuosity, and reduced sub-basal nerves in patients using glaucoma drops compared to healthy controls [66].

IVCM can also document changes in the cornea after glaucoma filtration surgery to evaluate for surgical success. For instance, preoperative DC density and goblet cell density (GCD) are correlated with filtration surgery outcomes [67]. These parameters were measured at the upper bulbar conjunctiva corresponding to the bleb site pre-operatively and at the bleb site postoperatively. Images were acquired from the epithelium and subepithelium (10–50 microns of depth). GCs may transport aqueous humor through the bleb wall [68] and DCs are the source of immune-regulatory cytokines [69], so increased GC and decreased DC are predictors of good outcomes. Hence, IVCM of the conjunctiva may represent an imaging tool to predict surgical success in glaucoma [67]. However, the study did not assess objective markers of dry eye, such as Schirmer’s test or TBUT. 

In addition, IVCM can be used to describe and compare the conjunctival filtering bleb features after XEN gel implantation and trabeculectomy, providing objective evaluation at a cellular level. For instance, IVCM was used to evaluate parameters like stromal meshwork reflectivity (SMR). As SMR represents an indirect indicator of the collagen content within the conjunctival stroma, a hyper-reflective pattern was a sign of collagen deposition, scarring, and potentially poorer clinical outcomes. After trabeculectomy, blebs showing a low degree of reflectivity and a thick wall are more likely to have a good filtering function [70]. However, this study did not evaluate the success or failure of these procedures in the long term as it only included blebs with a completely successful filtering function.

### 3.7. Corneal Graft Versus Host Disease (GVHD)

IVCM studies related to inflammation-related dry eyes from immune, toxic, or environmental causes are summarized in Table 6. For example, DED can also be mediated by severe immune reactions such as Graft-versus-host disease (GVHD), which is an inflammatory immune disease arising from an immunologic attack by donor alloreactive T cells that result in damage to vital organs, including the ocular surface of the eye [71].

Patients with ocular GVHD adjusted for ODSI and corneal staining displayed significantly decreased corneal epithelial cell density, SNP fiber density, and reflectivity compared to DED from other causes and healthy controls, while nerve tortuosity and epithelial DC density were increased in both oGVHD and DED groups [72]. This is in agreement with previous cross-sectional studies done [73,74,75]. As patients with DED unrelated to GVHD and ocular GVHD typically present with similar symptoms, IVCM could be used to evaluate and monitor patients with dry eyes due to GVHD and non-GVHD.

Patients with chronic GVHD had worse meibography scores, reduced corneal sub-basal nerve plexus densities, lower TBUT scores, lower Schirmer I values and higher corneal staining scores. There was extensive loss of meibomian glands in both superior and inferior eyelids. In patients with chronic GVHD, the ensuing long-term inflammation often results in fibrosis of the ocular surface and cicatrizing conjunctivitis [76]. Hence, patients with chronic GVHD are at high risk for developing DED and MG dysfunction [77]. It is unclear if the IVCM signs of GVHD are linked to the more severe MG dysfunction compared to the DED group.

### 3.8. Contact Lens-Related Conditions

Clinical studies using IVCM for contact lens-related problems are summarized in Table 7. Estimates of total contact lens (CL) wearers worldwide in 2005 were as high as 140 million and hence even complications with a low incidence may affect a large number of individuals [78]. While the majority of complications are minor such as conjunctival hyperemia and corneal edema from overwear, there are serious sight-threatening complications such as infectious keratitis [79]. IVCM of the central cornea observed a higher density of DCs in contact lens wearers compared with non–contact lens wearers. CL lens has been known to activate and increase DC, contributing to ocular surface inflammation and a decrease in SNP. This decrease in SNP has been hypothesized to be due to increased DC and activated inflammation [80]. This finding has also been confirmed in soft lens wearers [81].

The precise etiology of “corneal infiltrative events” (CIE) which arise during CL wear, including both corneal infections and noninfectious inflammatory events [82], is not well understood. The incidence of symptomatic CIEs during extended soft lens wear ranges from 2.5 to 6%; when asymptomatic CIEs are included, the incidence can be as high as 20–25% [83].

IVCM can thus be potentially used to assess the subclinical response of the ocular surface in CL wearer. The risk of developing CIEs is 12.5 times higher in reusable lenses (those stored overnight in disinfecting solution throughout their usage period, which is typically 2 weeks or 1 month) compared with daily disposable lenses [80]. Interestingly, DC density was higher in reusable lens wearers than in daily disposable CL wearers [82]. 

IVCM can also study changes in corneal nerves associated with contact lenses. Orthokeratology (OK) involves using specially designed and fitted GP contact lenses to reshape the corneal surface for the temporary correction of refractive error. Lenses are only worn at night during sleep and removed on waking to provide clear, unaided vision throughout the day. IVCM has found that nerve fiber density (NFD) is decreased in OK wear [84,85]. This reduced NFD is associated with reduced corneal sensitivity and increased nerve tortuosity as well [85].

## 4. Discussion

### 4.1. Limitations

Several studies were limited by their cross-sectional nature and hence inability to prove causation between the disease and IVCM parameters studied [14,38,52,55,56,61,70,77]. In addition, many studies were limited by their small sample size [14,24,55,61,66,68,70,77,80]. 

Some study designs include biases, with either no placebo group for comparison [37] or a lack of standardized treatment [68]. A study had no control groups or insufficient control (controls had dry eye symptoms) [52]. Others had study populations that may not be representative. For instance, in one study, most participants were female, and hence the results may not be extrapolated to the general population [24].

There were also limitations in the IVCM technique. A central area for analysis was selected to ensure consistent measurements across patient groups [66], but multiple scans in different areas could potentially give a more comprehensive assessment.

There may be significant inter-observer and intra-observer variability with poor repeatability and manual processing is laborious and time-consuming. Furthermore, IVCM can only image a very small field of view, hindering reproducible imaging of the same areas over time.

A limitation of our study was complying with all items for a systematic review. We did not register the protocol for this review in a review registry which is a flaw according to AMSTAR-2 (Supplementary Table S1). We have tried to reflect, in the material and methods section, the entire search protocol as it was carried out and the search strategy designed. For conducting a proper systematic review, out of sixteen questions, we answered “Yes” to nine questions, with partial “Yes” to one and “No” to five questions. We missed item 7 (justification for excluding individual studies—we did not provide the full list of excluded articles, but this can be made available on request), item 10 (funding sources of papers as the paper would be too lengthy), item 11, 12, and 15 as these questions are not relevant when meta-analysis are not performed. We have a partial “Yes” to item 4 (we searched only 1 database instead of 2, but we have provided the keywords and search strategy used).

However, this review is about an imaging diagnostic tool, IVCM, not about specific therapy. As there were no studies that evaluated health outcomes based on the use of IVCM vs. the lack of such imaging, the AMSTAR2 and ROB2 could only be applied to study outcomes of specific treatment interventions.

### 4.2. Future Research Directions

Due to the high cost of this technology, the widespread deployment and use of IVCM in clinical practice are limited. Hopefully, improvement in hardware and wider use may bring the cost down. Analysis of corneal SNP on IVCM images can be fully automated, semi-automated, or manual. The fully automated technique requires no manual input from the observer and is faster and more suited for large trials and longitudinal studies requiring analysis of a large number of images. However, there might be more false-negative and false-positive errors that require improvement of these algorithms in the future [86].

As mentioned earlier, one of the drawbacks of IVCM is its small field of view, preventing an overview of SNP architecture and necessitating subjective image sampling of small areas of the SNP for analysis. Hence, future directions also include large-area imaging and mapping or mosaic technique. Corneal SNP can be reconstructed by automated mosaicking, with an average mosaic image size corresponding to 48 individual IVCM fields of view [87].

The use of artificial intelligence (AI) and increasing automation will improve the speed and accuracy of image analysis. Freeware, including ImageJ (NIH) and image processing packages for python (e.g., scikit-image, OpenCV) and others, have many built-in functions which allow for custom scripting. Future advances are likely to include advances in machine-learning algorithms, which are currently making their way into commercial software packages [88]. High-speed networks will also improve the ease of using IVCM images in digital medicine.

## 5. Conclusions

IVCM is useful as a complementary technique for clinical diagnosis in various ocular surface disorders related to dry eye. With advances in hardware and software in the near future, it has the potential for further practical impact and can be used for a multitude of OSD for diagnosis, management, and prognostication.

## Figures and Tables

**Table 1:** Studies on Meibomian Gland Dysfunction (MGD).

Area of Study	Authors	Main Contribution to Literature	Design	Sample Size (Eyes)	Source of Participants	No. of Visits	HRT	Main Outcomes	Main Findings	Limitations
Immune cellular metrics of inflammation	Qazi Y.	Only study which uses IVCM-based immune-cellular metrics to assess the inflammation of MGD.	Cross-sectional, retrospective	29	Outpatient	1	HRT III-RCM	Density of immune cell, epi, or stromal No. of periglandular immune cell Area of IGIC Luminal width, length, and thickness of internal luminal hyperreflective ring in ductules	EIC and IGIC increased in highly symptomatic patients that have minimal corneal staining and correlate with symptoms and clinical signs.	A cross-sectional study does not demonstrate that the progression of MGD causes increases in immune cells. Small sample size.
Evaluation of corneal layers in MGD	Azizi S.	Evaluating individual corneal layers using IVCM	Single-center, prospective	92	Outpatient	1	HRT III-RCM	Surface area and cell coverage of basal epithelial anterior keratocytes, posterior keratocytes, and endothelial cells. Stromal nerve thickness	Basal epithelial cell density reduced, the area increased, and stromal nerve thickness greater in the MGD group.	Study repeats the older methodology (no new findings)
Classification in the diagnosis of MGD	Randon M.	Using IVCM as a new way to classify the various pathophysiological system for MGD	Cross-sectional, retrospective	115	Outpatient	1	HRT II-RCM	Combination of grades of meibum reflectivity, intraepithelial/interglandular inflammation, glandular fibrosis	Strong correlation between IVCM score (Type 0 normal MG; type 1 obstructed MG without inflammation or fibrosis; type 2 MG inflammation without fibrosis; type 3 MG fibrosis), and meibography score.	Study about the grading severity of MGD needs more studies to confirm accuracy.
MGD with different symptoms	Zhao H.	Attempted to differentiate symptoms of MGD using IVCM	Cross-sectional, retrospective	60	Outpatient	1	HRT II-RCM	MG acinar unit density palpebral conjunctival inflammatory cell density periglandular inflammatory cell density MG acinar unit area MG acinar unit longest diameter MG acinar unit shortest diameter	More severe symptoms had more significant fibrosis and a severe decrease in the size of MG acinar units. DED symptoms negatively correlated with confocal microscopic parameters and positively correlated with conjunctival inflammatory cells and Langerhans cells	Unable to test MG lipid and inflammatory factors which could contribute to DED.

DED: dry eye disease; EIC: epithelial immune cell; IGIC: intraglandular immune cell; IVCM: in vivo confocal microscopy; MGD: meibomian gland dysfunction; HRT: Heidelberg Retinal Tomograph; RCM: Rostock Cornea Module.

**Table 2:** Studies on Sjogren Syndrome (SS) and Non-Sjogren Syndrome Dry Eye (NSSDE).

Area of Study	Authors	Main Contribution to Literature	Design	Sample Size	Source of Participants	No. of Visits	HRT	Main Outcomes	Main Findings	Limitations
Imaging of POV in DED	Ghouali	In all quadrants, fewer POVs were detected in DED patients than in normal subjects	Prospective case-control	163	Outpatient	1	HRT II-RCM	POV found predominantly in superior (*p* < 0.001) and inferior (*p* < 0.001) quadrants compared to nasal and temporal quadrants	En-face SD-OCT showed POV as a radially oriented network located in the superficial corneoscleral limbus, with a good correlation with IVCM features	Depth of analysis (70 μm below the corneal/conjunctiva surface) might not capture the entire POV structure.
Corneal Sub-basal Nerve Plexus in DED (New Fully Automated System)	Giannaccare	ACC Metrics detected SNP alterations in DED, good diagnostic performance in discriminating DED.	Cross-sectional	69	Outpatient	1	HRT-RCM	CNBD CNFL CNFW	Lower CNBD, CNFL & higher CNFW in DED compared to controls	Small sample size ACC metrics cannot analyze and quantify nerve tortuosity, a well-recognized metric affected in DED
DED and Low Sub-basal Nerve Density and Corneal Endothelial Cell Loss	Kheirkhah	DED associated with accelerated corneal endothelial cell loss	Retrospective 33.2 ± 10.2 months	40	Outpatient	2 (baseline, next visit)	HRT III-RCM	Densities of corneal endothelial cells and sub-basal nerve	Initial visit DED: lower densities of corneal endothelial cells and sub-basal nerves than control Endothelial cell loss negative correlation with initial sub-basal nerve density	Retrospective design, small sample size, did not evaluate the morphology of endothelial cells Only central CECD was measured.
Evaluation of Objective Visual Quality in Dry Eye Disease and Corneal Nerve Changes.	Ma, Jiahui	Longer and wider corneal nerves were associated with better objective visual quality in DED	Prospective study	98	Outpatient	1	Not specified	CNFL, objective scatter index, mean objective scattering index, modulation transfer function, Strehl ratio.	Patients with longer and wider corneal nerves had better objective visual quality	No control group. Changes in Langerhans cells not summarized. Small sample size
Quantification of Corneal Sub-basal Nerve Tortuosity in DED and Its Correlation With Clinical Parameters	Ma, Baikai	New parameter: Aggregated measure of tortuosity (Tagg) for quantification of corneal sub-basal nerve tortuosity.	Cross-sectional case-control	49	Outpatients	1	RCM	Tagg higher in DED than controls (*p* < 0.001). Tagg correlated with OSDI (r = 0.418) & negatively correlated with BUT	Higher Tagg may be linked to ocular discomfort, visual function disturbance, and tear film instability	Excluded images with DCs and obvious neuromas because DCs co-segmented with nerves
Corneal Sub-basal Nerve Analysis in DED and Clinical Correlations	Liu Yan	IVCM is a useful tool to evaluate corneal changes in DED, such as in sub-basal nerves and Langerhans cells (LCs)	Cross-sectional study	107	Outpatient	1	HRT II-RCM	CNFL CNFT CNFW	Langerhans cells no. not correlated with symptoms. CNFL negatively correlated with sensitivity to light; CNFW positively correlated with OSDI, pain, blurred vision; CNFT positively correlated with sensitivity to light	No healthy controls. Cross-sectional study.
IVCM in Primary Sjögren Syndrome and Sicca Syndrome Patients	Joana C.	Using IVCM to diagnose and differentiate immune-mediated DED from other forms of DED	Cross-sectional, retrospective	136	Outpatient	1	HRT III-RCM	SNP Nerve density Nerve length Nerve tortuosity	pSS and non-SS sicca pts had lower corneal SNP plexus density and length, increased tortuosity compared to healthy controls, unable to differentiate between pSS and non-SS	Some patients in immune-mediated DED groups did not meet the criteria for DED.
Ocular Surface Alterations in Patients With Fibromyalgia	Turan E.	First study to evaluate corneal microstructures in FM.	Cross-sectional, retrospective	76	Outpatient	1	HRT III-RCM	Basal epithelial cell density SNP density SNP tortuosity	Basal epithelial cell density, total nerve density, long new fibers, total no. of nerves lower in patients with FM.	Small sample size.
IVCM in SSDE	Michele L.	Using light backscattering as a parameter in IVCM to evaluate SSDE	Cross-sectional, retrospective	110	Outpatient	For 6 cont. months	HRT III-RCM	Light backscattering	LB is higher in patients with SSDE, which is postulated to be due to higher levels of inflammation in SSDE.	Cross-sectional study does not prove causation.
Corneal epithelium in SSDE vs. NSDE	Olivia L.	Assessing reproducibility and reliability of other studies on the same topic.	Cross-sectional, prospective	78	Outpatient	1	HRT III-RCM	Superficial, basal, outer wing, and inner wing epithelial cell density	Superficial epithelial, inner wing, and basal cell density are lower in SSDE and NSDE compared to control. No diff in SSDE and NSDE. Outer wing cell density lower in SSDE compared to NSDE and control, no sig difference	Cross-sectional study does not prove causation.
Cornea nerve structure with primary SSDE vs. NSDE	Fangting L.	Evaluate IVCM morphology of corneal SNP and its relationship with clinical parameters	Cross-sectional, prospective	42	Outpatient	1	HRT III-RCM	SNP Nerve density Length, max length Number Tortuosity	SS lower density, no. of nerves than NSDE Greater nerve tortuosity in SSDE than NSDE Mean length and max length similar in both	Small sample size, area selected for IVCM analysis is not representative of the whole plexus
Tear lacritin levels in patients with SSDE	Nancy M.	First study to inv association between tear lacritin levels and SS patients	Cross-sectional, prospective	20	Outpatient	1	Nidek Confoscan 4	SNP Nerve fiber density Nerve fiber length Nerve branch density Tortuosity	Nerve fiber density and length sig decreased in SSDE No mention of branch density or tortuosity.	Small sample size, focus of study is not on IVCM
Corneal Innervation, Inflammation, and Symptoms in DED	Tepelus	NSDE and SSDE: alterations in corneal innervation and increased DCs. Corneal nerve density and reflectivity are correlated with OSDI.	Prospective case-control study	78	Outpatient	1	HRT III-RCM	CNBD CNFT, Reflectivity of corneal nerves, CDCD	CNBD decreased in SSDE & NSDE, Increased CNFT & decreased reflectivity in both. DCs increased in SSDE & NSDE compared to controls Correlations between DNF & DC (r = −0.57), between DNF & OSDI (r = −0.91) and between RNF & OSDI (r = −0.75).	Relatively small no. of patients Topical and systemic corticosteroids have a potential effect on epithelial DC density and OSDI Most participants females—results may not be extrapolated to male patients.
Proinflammatory Markers, Chemokines, and Enkephalin in DED	Pierre Nicolle	DED patients have significantly higher corneal DC density compared to controls	Prospective case-control study	47	Outpatient	1	HRT-RCM	Sub-basal nerve density CDCD	Sub-basal nerve density was significantly lower in DED compared to controls; DED patients had a significantly higher corneal DC density compared to controls.	Not all participants had IVCM in both eyes. Low quantity of mRNA in some impressions. No protein level markers nor immune composition.
Correlation of Corneal Immune Cell Changes with Clinical Severity in DED	Aggarwal	DC density and morphology correlated with DED severity, DC density increased in mild DED, morphological changes in severe DED.	Retrospective, cross-sectional	349	Outpatient	1	HRT III-RCM	DCD Size Morphology	DC density is higher in DED compared to controls. Morphologically, the number of dendrites, DC size, and field were significantly larger in DED than in controls.	Retrospective. Only analyzed central corneal images.
Tear Nociception-Associated Factors, Cornea DCD in DED	Khamar	Altered tear fluid soluble factors associated with ocular surface discomfort, TBUT, Schirmer’s test, and cornea DCD	Case-control cross-sectional	80	Outpatient	1	HRT II-RCM	Cornea DCD, SNP features	Cornea DCD is significantly higher in DED patients. No significant difference was observed in SNP features.	Cross-sectional design

CNBD: Corneal nerve branch density; CNFL: Corneal nerve fiber length; CNFT: corneal nerve fiber tortuosity; CNFW: corneal nerve width; DC: dendritic cell; DCD: dendritic cell density DED: dry eye disease; HRT: Heidelberg Retinal Tomograph; LCs: Langerhans cells; POV: Palisade of Vogt; OSDI: ocular surface disease index; RCM: Rostock Cornea Module; SD-OCT: spectral-domain optical coherence tomography; SNP: sub-basal nerve plexus; SSDE: Sjogren’s Syndrome Dry Eye; TBUT: tear break-up time.

**Table 3:** Studies on Treatment-Induced Changes in Dry Eye.

Area of Study	Authors	Main Contribution to Literature	Design	Sample Size	Source of Participant	No. of Visits	HRT	Main Outcomes	Main Findings	Limitations
Corneal SNP density in SSDE treated with cyclosporin A	Ora L.	Evaluate SNP changes in SSDE treated with cyclosporin A	Longitudinal, prospective, observational	45	Outpatient	2 (base, 6 months)	HRT III-RCM	SNP density, number, reflectivity, tortuosity DC density	SNP density sig increased after CsA, a/w decreased tortuosity, and DCs number	Lack of placebo group and repeated IVCM in healthy group 6 months later.
2-month treatment of CBS eye drops in ocular surface disease	Giannaccare G.	Reported corneal cell morphology and corneal nerves after CBS therapy	Prospective, observational, cross-sectional	20	Outpatient	1	HRT III-RCM	Giant epithelial cells SNP number and tortuosity Neuromas, beading, and DCs in the central cornea.	Higher total nerve number and lower tortuosity Giant epithelial cells, beading, and neuromas decreased. DC density unchanged	Cause of DED in 20 patients was different
DED treated with different sources of homologous eye drops	Giannaccare G.	RCT to investigate the difference in the effect of allo-PBS and CBS eye drops on corneal nerve morphology	Randomized, double-blinded RCT	30	Outpatient	2 (base, 30 days)	HRT III-RCM	CNFD CTBD CNFL CTBD CNFA CNFW CNFrD	Overall CNFD, CNFL, CNFrD sig increased CNFW decreased CNFrD increase higher in CBS eye drops than in allo-PBS	Small sample size, follow-up time too short to draw a conclusion on eye drop efficacy
Patients with DED treated with topical cyclosporin	Iaccheri B.	No studies have yet evaluated topical cyclosporine on IVCM parameters in DED	Prospective, observational, cross-sectional	42	Outpatient	4 (base, 1, 3, 6 months)	HRT II-RCM	SNP density, number, tortuosity, reflectivity Activation of stromal keratocytes	Density of intermediate epithelial cells increased Keratocyte activation decreased. SNP number, reflectivity, and tortuosity decreased	Small sample size, causes of DED in the patient group, not constant
Mild DED Trial with artificial tears or steroids and relationship to corneal DCs	Li Bei	Topical steroids can reduce corneal DCs	Case-control	72	Outpatient	1	Not specified	Tear and conjunctival cytokines, amount of DCs	More DCs in cornea epithelium of dry eye s with symptoms outweighing signs than common mild dry eye and control groups. After glucocorticoid treatment, the number of DCs significantly decreased	IVCM findings are limited to DCs
Efficacy of 2% Topical Rebamipide on Conjunctival Squamous Metaplasia and Goblet Cell Density in DED	Simsek	Topical use of 2% rebamipide for 3 months associated with improvements in ocular surface differentiation due to mucosal changes	Prospective interventional study	15	Outpatient	2 (baseline, 3 months)	HRT II RCM	Evaluation of nucleocytoplasmic ratios and corneal ECs	Significant improvements in mean corneal epithelial cell density and nucleocytoplasmic ratios after treatment.	Small sample size
Omega-3 on corneal nerves in DED	Chinnery H.	RCT to investigate the effect of omega-3 on nerve parameters in DED	Randomized, double-blinded, RCT	12	Outpatient	2 (base, 90 days)	HRT III-RCM	CNFD, CNFL, CNBD, CTBD, CNFW, and CNFA Hyperreflective DCs	CTBD and CNBD increased after 90 days of omega-3 Sig crossover interaction for CNFL CNFA, CNFD, CNFW no difference	Small sample size
IVCM in DED after autologous eye drop	Mahelkova G.	Added to the current literature of IVCM findings after topical therapies on DED	Prospective, observational, cross-sectional	26	Outpatient	2 (base, 3 month)	SSCM, Confoscan 3, NIDEK Technologie, Padua, Italy	Superficial and basal epithelial cell density Density of keratocytes Density of epithelial cells. SNP total nerve length, number, number of branches, tortuosity DC density	Basal EC density decreased sig Sup EC, no. of DCs, activated keratocytes did not change sig. No differences in the other corneal cell layers or in the status of the nerve fibers	Small sample size, no control group to compare against
Combined hyaluronic acid (HA) and coenzyme Q10 eyedrops vs. HA alone eyedrops in DED	Postorino E.	Added to IVCM findings in the dry eye after novel medication (XLHA and CoQ10)—the study of epithelial cell reflectivity, keratocytes, stromal matrix parameters	Randomized, single-blinded RCT	40	Outpatient	4 (base, 15, 30, 90 days)	Confoscan 4 confocal microscope (Nidek Technologies)	Hyperreflectivity of ECs Morphological features of keratocytes Stromal matrix	EC hyperreflectivity decreased in the combination group Sig improvement in keratocyte and stromal matric in the combination group	Small sample size
Ocular nebulization of Vitamin B12 vs. oxytocin in DED	Yang J.	First to report IVCM changes after nebulization in ophthalmology	Randomized, double-blinded RCT	38	Outpatient	3 (base, 1, 3 month)	HRT III-RCM	Basal epithelial cell density Sub-basal DC density Nerve density Nerve tortuosity	Basal EC and SNP density increased, DC density decreased in all groups Tortuosity no change Higher EC density at 3 months, lower DC density at 1 month in the B12 group	Ages in both groups different, no control group

CBS: cord blood serum; CNBD: corneal nerve branch density; CNFA: corneal nerve fiber area; CNFD: corneal nerve fiber density; CNFL: corneal nerve fiber length; CNFrD: corneal nerve fractal dimension; CNFW: corneal nerve fiber width; CTBD: corneal nerve fiber total branch density; DC: dendritic cells; DED: dry eye disease; EC: epithelial cell; HRT: Heidelberg Retinal Tomograph; IVCM: in vivo confocal microscopy; PBS: peripheral blood serum; RCM: Rostock Cornea Module; RCT: randomized controlled trial; SNP: sub-basal nerve plexus; SSDE: Sjogren’s syndrome Dry Eye.

**Table 4:** Studies on Systemic Disease-related Dry Eye.

Area of Study	Authors	Main Contribution to Literature	Design	Sample Size	Source of Participants	No. of Visits	HRT	Main Outcomes	Main Findings	Limitations
Corneal nerve alterations in children and youths with T1DM	Tiziano C	Early signs of corneal nerve degeneration were found in children and youths with T1DM	Retrospective case-control study	201	Outpatient	1	HRT III-RCM	CNFL CNFD CNBD CTBD CNFrD	All IVCM parameters, except CTBD, were significantly lower in the T1D patients. Glycometabolic control (HbA1c, visit-to-visit HbA1c variability, and mean HbA1c) and blood pressure were inversely correlated with IVCM parameters.	Small sample size; sample had European ancestry, so results cannot necessarily be extended to children and adolescents with other ethnic backgrounds.
Reduced Corneal Nerve Fiber in T2DM	Neil S Lagali	Wide-area mosaic images provide reference values for mosaic CNFL (mCNFL) and whorl CNFL (wCNFL) and reveal a progressive degeneration of the SBP with increasing duration of type 2 diabetes.	Population-based study	163	Outpatient	1	HRT III-RCM	mCNFL Apical wCNFD	mCNFL in T2DM reduced relative to non-diabetic subjects Lower mCNFL is associated with diabetes and increased HbA1c levels Apical wCNFD was unaffected by diabetes or HbA1c Global SNP patterns revealed marked degeneration of secondary nerve fiber branches outside the whorl region in long-duration diabetes.	Small sample size
DC maturation in corneal epithelium associated with TNF receptor superfamily member 9	Neil S Lagali	Develop a non-invasive means to monitor the status of inflammatory DC subsets in the corneal epithelium as a potential biomarker for the onset of inflammation in T2DM	Cohort study	81	Outpatient	1	HRT III-RCM	Quantification of DCs	With the onset of diabetes, the proportion of mature, antigen-presenting DCs increased and became organized in clusters. TNF receptor superfamily member 9 (TNFRSF9) is associated with the observed maturation of DCs from an immature to mature antigen-presenting phenotype.	Small cohort size, narrow focus on the relationship between systemic markers of inflammation and corneal DCs
Sub-basal nerves in wide-area corneal nerve plexus mosaics in T2DM	Reza A Badian	Sub-basal nerve degeneration in T2DM can vary according to anatomic location	Cross-sectional study	163	Outpatient	1	HRT III-RCM	SNP CNFL	In long-term T2DM, nerve density in the left superior sector of SNP decreased while that in the central superior SNP increased relative to healthy subjects with normal glucose tolerance CNFL is not affected by diabetes	Cross-sectional nature
Imaging of Corneal Sub-basal Whorl-like Nerve Plexus	Tsugiaki Utsunomiya	IVCM measurements of whorl-like patterns may accurately define the extent of corneal nerve damage to monitor diabetic peripheral neuropathy.	Observational study	68	Outpatient	1	HRT III-RCM	CNFL	Total CNFL is significantly shorter in DM group than in the control group and decreases with the progression of diabetic retinopathy, nephropathy, neuropathy, and decreased corneal sensation.	Small no of patients, captured a whorl-like pattern in half of the subjects (visual fixation unstable with fatigue)’ T1DM and T2DM subjects combined with analysis
IVCM of Corneal Nerves: Ocular Biomarker for Peripheral and Cardiac Autonomic Neuropathy in T1DM	Stuti L Misra	Correlation of corneal SNP density with total neuropathy score suggests that reduced corneal nerve density reflects peripheral neuropathy in diabetes.	Case-control study	93	Outpatient	1	HRT II-RCM	Corneal SNP density Corneal sensitivity	Corneal SNP density and corneal sensitivity were significantly lower in diabetes compared to controls. A modest negative correlation between total neuropathy score and SBN density was observed.	Sub-basal nerve branching and tortuosity were not considered nor analyzed
Epithelial changes with corneal punctate epitheliopathy and correlation with time to healing in T2DM	Jing-Hao Qu	Increased LC and decreased SNP in T2DM with corneal punctate epitheliopathy	Retrospective study	160	Outpatient	1	HRT III-RCM	Density of BEC, SNP, and LC	LC density, SNP density, and BEC density were reduced in the T2DM group compared with controls. LC density in the T2DM group showed a negative correlation with SNP density. SNP density in the T2DM group showed a positive correlation with BEC density. BEC density in the T2DM group showed a negative correlation with healing time.	IVCM images only from the first patient visit and no post-treatment images for comparison. Glycemic control data was not collected in T2DM patients.
Association between alterations of corneal SNP and long-term glycemic variability	Marco P	HbA1c and disease duration were independent predictors of damage to SNP in T1DM.	Consecutive cross-sectional study	40	Outpatient	1	HRT-RCM	CNFD CNFL CNFrD CTBD CNFA CNFW	Diabetes duration and all-time SD of HbA1c were independently associated with CNFD, CNFL, and CNFrD. Analysis of the association among IVCM parameters and specific subtypes of diabetic neuropathy showed that altered cold sensitivity was independently associated with CNFD.	Small no of patients, lack of longitudinal IVCM analysis, included only T1DM
Ocular and Cutaneous Rosacea	Liang, Hong	IVCM features of rosacea patients combined with quantification of *Demodex*	Case-control cross-sectional study	44	Outpatient	1	HRT-RCM	MG (IVCM-MG) cheek (IVCM-Cheek) alterations, *Demodex counts:* IVCM-MG-Dex IVCM-Cheek-Dex	IVCM-MG correlated with IVCM-Cheek IVCM-MG-Dex correlated with IVCM-Cheek-Dex	cannot image deeper structure no normal controls
Sub-basal Nerve Plexus Changes in Chronic Migraine	Shetty, Rohit,	Changes in SNPP support the role of the trigeminal system in the pathogenesis of ocular symptoms in migraine	Cross-sectional study	84	Outpatient	1	HRT-RCM II	CNFD NFL CNBD CTBD CNFA Average CNFW	SNPP: a significant decrease in CNFL, CTBD, CNBD, and CNFA in migraine with photophobia	Changes during ictal period not done Small sample
Chinese TAO	Wu LQ	Abnormal corneal sub-basal nerves observed in active and inactive Chinese TAO	Cross-sectional study	58	Outpatient	1	HRT III-RCM	CNFD CNBD CNFL CTBD CNFA CNFW ACNFrD	SNP parameters of TAO decreased compared to controls; correlations between CNFD, CNBD, CNFL, CTBD, CNFA, and ACNFrD	Small sample size No adjustment for dry eye or tear function
Meibomian Glands structure in Graves’ Orbitopathy	Cheng S	IVCM found obstruction and inflammation in MG of GO patients	Cross-sectional observational study	142	Outpatient	1	HRT III-RCM	MAD, MALD, MASD MOA, MAI, MSR, AWI, API, MG fibrosis.	Compared to controls, GO: lower MOA, MAD; greater MALD, MASD, MAI, MSR, and MG fibrosis Active GO: higher MAI, AWI, & API, Inactive GO: higher MSR and MG fibrosis GO: AWI and API positively correlated with CAS, MG fibrosis negatively correlated with CAS.	Control group is not representative of the healthy population—some had dry eye symptoms and inadequate MG performance
Corneal changes in Mucous Membrane Pemphigoid (MMP)	Tepelus	Microstructural corneal changes in MMP	Prospective single-center cross-sectional study	40	Outpatient	1	HRT III-RCM	Morphology of corneal epithelial layers, stroma, and endothelium, corneal nerves, and presence of DCs	Decreased corneal nerve density and elevated DC in non-end-stage MMP compared with controls.	Small sample size
Corneal Nerve in Parkinson’s Disease.	Misra, Stuti L.	Significant reduction in corneal SNP density in Parkinson’s, which is associated with cognitive dysfunction	Cross-sectional study	30	Outpatient	1	HRT II-RCM	Corneal SNP density	Corneal SNP density markedly reduced in Parkinson’s compared with controls	Limited sample size mild disease excluded

API: acinar periglandular interstices inhomogeneity; AWI: acinar wall inhomogeneity; BEC: basal epithelial cell; CNFrD: corneal nerve fiber fractal dimension; CNBD: corneal nerve branch density; CNFA: corneal nerve fiber area; CNFD: corneal nerve fiber density; CNFL: corneal nerve fiber length; CNFW: corneal nerve fiber width; CTBD: corneal nerve fiber total branch density; DC: dendritic cell; Hba1c: hemoglobin A1c; LC: Langerhans cells; MAD: MG acinar density; MAI: MG acinar irregularity; MALD and MASD: MG longest and shortest diameters; MOA: MG orifice area; MSR: meibum reflectivity; SD: standard deviation; SNP: sub-basal nerve plexus; TAO: Thyroid-Associated Ophthalmopathy; TNF: tumor necrosis factor; T1DM: Type 1 Diabetes Mellitus; T2DM: Type 2 Diabetes Mellitus.

**Table 5:** Studies on Glaucoma-Related Dry Eye.

Area of Study	Authors	Main Contribution to Literature	Design	Sample Size	Participants	No. of Visits	HRT	Main Outcomes	Main Findings	Limitations
Structural Imaging of Conjunctival Filtering Blebs in XEN Gel Implantation and Trabeculectomy	Sacchi	First paper to use IVCM on epithelial cysts and a hypo-reflective bleb wall	Retrospective, cross-sectional, observational study	52	Outpatient	2 (baseline, 6 months)	HRT III-RCM	MMD, MMA, SMR	MMA and SMR values were lower in the XEN gel implantation compared with trabeculectomy	Retrospective, small sample size, inclusion of only completing successful filtering blebs
Prospective, Masked, 36 Months Study on Glaucoma Patients Medically Treated with PF or Preserved Monotherapy	Rossi	PF-tafluprost formulation does not alter corneal structures after 36 months of topical daily therapy	Prospective, Masked Study	93	Outpatient	7 (Baseline and every 6 months for 3 years)	Confoscan 4 (Nidek technologies)	Activation of keratocytes, number of sub-basal plexus nerve fibers, tortuosity, number of bead-like formations, endothelial cellular density.	At baseline, keratocyte activation similar in the 3 groups Over months, naïve patients treated with PF-tafluprost reduced keratocyte activation. Sub-basal nerves increase in patients switched to PF-tafluprost	Investigation limited to the center of the cornea, different results were obtained by inspecting the corneal periphery Limited sample size
Long Term Safety and Tolerability of Tafluprost 0.0015% vs. Timolol 0.1% Preservative-Free in Ocular Hypertensive and in Primary Open-Angle Glaucoma Patients: A Cross-Sectional Study	Rolle, Teresa	Both therapy: show alterations in corneal microstructure but no side effects on tear function.	Retrospective, single-masked, observational, cross-sectional study	108	Outpatient	1	HRT II-RCM	Basal EC density Stromal reflectivity (keratocytes activation) No. sub-basal nerves, Sub-basal nerve tortuosity, Sub-basal nerve reflectivity, Endothelial cell density.	Tafluprost: higher OSDI score, basal EC density, stromal reflectivity, sub-basal nerves tortuosity, and less number of sub-basal nerves than control Timolol: higher OSDI, basal EC density, stromal reflectivity, and sub-basal nerve tortuosity, less no. of sub-basal nerves than controls.	Only examine the central cornea, Retrospective nature
IVCM of Conjunctiva as a Predictive Tool for the Glaucoma Filtration Surgery Outcome	Mastropasqua	Preoperative DCD, GCD, SMR are parameters correlated with filtration surgery outcome, with DCD presenting the strongest correlation	Prospective, single-center, case-control	81	Outpatient	2 (baseline, 12 months)	HRT III-RCM	Conjunctival DCD, GCD, SMR	12 month IOP reduction negatively correlated with baseline DCD and SMR and positively with GCD IVCM of the conjunctiva may represent an imaging tool to predict the surgical success in glaucoma.	SMR is arbitrary. Unsure if DCD, GCD, and SMR are different before therapy between groups. Possible normal interindividual variability in DCs and GCs, and in the stromal density of conjunctiva
Uveo-Scleral Outflow Pathways after UCCC in Refractory Glaucoma	Mastropasqua	UCCC induced modifications of sclera and conjunctiva structures	Prospective interventional, case-control study	44	Outpatient	2 (baseline, 1 month)	HRT III-RCM	Area of conjunctival microcysts (MMD: cysts/mm^2^; MMA: µm^2^) at IVCM	MMA and MMD increased in both groups of UCCC (4 s and 6 s), with values higher in 6 s UCCC	Cases and controls differ because controls did not have refractory glaucoma due to ethical concerns; Did not evaluate the intra-subject agreement.
In Vivo Distribution of Corneal Epithelial DCs in Medically Controlled Glaucoma Patients (MCGP)	Mastropasqua	DCs increase in the entire cornea, with a higher density at the limbus, may induce glaucoma-related ocular surface disease	Retrospective observational study	80	Outpatient	1	HRT III-RCM	Limbal and central DC density, DCs morphology and distribution.	DC density is higher in glaucoma & DED than in controls DC density is higher in patients taking preserved than patients taking PF drops DC density correlated with staining	Retrospective nature; Did not investigate other corneal features such as sub-basal nerve plexus or the superficial epithelial layers.
The Ocular Surface after Successful Glaucoma Filtration Surgery	Agnifili	Whole ocular surface system objectively improved after completely successful glaucoma filtration surgery.	Prospective case-control study	54	Outpatient	2 (baseline, at 6 months)	HRT III-RCM	GCD, limbal DCD, SCNI; MGD, MGI, and HLA-DR positivity.	At the 6th month, surgical group: GCD increase, and limbal DCD, SCNI, MGI, HLA-DR, OSDI decrease OSDI correlated with GCD, MGI, SCNI, limbal DCD, & HLA-DR	Cannot ascertain whether changes are due to drug discontinuation, topical steroids, or both. Did not analyze structural corneal nerve parameters; MMC has a cytotoxic effect No MMC-independent glaucoma surgery as a comparison.
Meibomian Gland Features and Conjunctival Goblet Cell Density in Glaucomatous Patients Controlled With PTFCs	Agnifili	PTFCs were less toxic towards MGs and goblet cells compared with the L + T unfixed combination, with PF-BTFC presenting the most tolerated profile.	Case-control cross-sectional study	90	Outpatient	1	HRT III-RCM	MMAD, MMAA, InI, InAW, GCD	IVCM documented lower GCD, MMAD, and MMAA, and greater InI and InAW in glaucoma patients compared with controls.	Cross-sectional study Cannot provide MG and GC status before initiation of therapy. Possible unreported/transient ocular surface problems. Limited sample and for grouping.
Conjunctival GCs in Medically-Controlled Glaucoma	DI Staso	Glaucoma therapy leads to a marked reduction of GCs	Case-control, cross-sectional, non-interventional study	72	Outpatient	1	HRT III-RCM	GCD	GCD was reduced in both glaucoma groups and those with DED compared to healthy controls, markedly lower in group 2 compared to group 1. GCD was not different between DED and group 2. Negative correlation between GCD with OSDI and with TBUT	Did not allow evaluation of the racial differences in the GC population. Baseline GC before therapy unavailable, Did not consider patients controlled with three medications.

DCD: dendritic cell density; DED: dry eye disease; EC: epithelial cell; GC: goblet cell; GCD: goblet cell density; InAW: inhomogeneity of acinar wall (InAW); InI: inhomogeneity of glandular interstice; MCGP: medical controlled glaucoma patients; MMC: mitomycin-C; MMD: mean microcyst density; MMA: mean microcyst area; MMAA: mean Meibomian acinar area; MMAD: Mean Meibomian acinar density; MG: meibomian gland; MGD: meibomian gland density; MGI: meibomian gland inhomogeneity; PF: preservative-free; PTFCs: Prostaglandin/Timolol Fixed Combinations; OSDI: ocular surface disease index; SCNI: sub-basal corneal nerve inhomogeneity; SMR: stromal meshwork reflectivity; TBUT: tear break-up time; UCCC: Ultrasonic Cyclocoagulation.

**Table 6:** Studies on Inflammation-Related Dry Eye.

Area of Study	Authors	Main Contribution to Literature	Design	Sample Size	Participants	No. of Visits	HRT	Main Outcomes	Main Findings	Limitations
DED with and without chronic GVHD	Kheirkhah A	Only study concludes that symptomatology may be linked to local disease rather than the underlying systemic disease.	Retrospective, cross-sectional	52	Outpatient	1	HRT III-RCM	Corneal epithelium DC density Corneal sub-basal nerves Conjunctival EICs	No significant differences in IVCM parameters for both groups.	GVHD group treated with anti-inflammatory medications, lowering inflammatory changes.
Corneal features in ocular GVHD	Tepelus TC	IVCM revealed distinct microstructural changes in the corneas of patients with oGVHD and DED	Cross-sectional, observational	33	Population	1	HRT III-RCM	Epithelial cell density SNP (nerve density, tortuosity, reflectivity) DC density	Superficial EC density, basal cell density are lower in oGVHD and DED groups, with a significant difference in the former results (oGVHD lower). Nerve fiber density and nerve reflectivity were higher in decreased in oGVHD only.	Cross-sectional study cannot prove causation. Limited patients in the oGVHD group. Treatments not standardized
Association between Meibomian Gland Atrophy and Corneal Sub-basal Nerve Loss in Chronic Ocular GVHD	O Dikme tas	Patients with chronic GVHD are at high risk for developing DED and MG dysfunction. In chronic GVHD-related DED, MG loss does not appear to be a significant factor for corneal sub-basal nerve damage.	Cross-sectional	50	Outpatient	1	Confoscan 4, Nidek, Japan	Corneal sub-basal nerve densities Meibography scores	Chronic GVHD had worse meibography scores, reduced corneal sub-basal nerve plexus densities, lower TBUT scores, lower Schirmer I values and higher corneal staining scores Corneal sub-basal nerve densities of patients with GVHD did not correlate with meiboscores but showed a weak correlation with Schirmer I test values.	Small sample size No separate group for non-GVHD dry eye patients.
Face Mask-Related Ocular Surface Modifications During COVID-19 Pandemic	Mastropaqua	The use of FM increases ocular surface inflammation and negatively impacts the quality of life in patients with DED.	Prospective	128	Outpatient	2 (baseline, 90 days)	HRT-RCM	CDCD GCD	DCD significantly increased in prolonged wear, whereas GCD did not significantly change.	No controls not using FMs. Short-term study Long-term studies may reveal FM-related GCD changes.
Ocular Surface, Meibomian Gland Alterations, and Cornea changes in Chronic Cigarette Smokers.	Ağın	Corneal nerve changes are found in chronic smokers. Smoking has an adverse effect on ocular surface parameters	Cross-sectional case-control	100	Outpatient	1	Confoscan 3.0 (Nidek)	Basal EC density, anterior and posterior keratocytes, endothelial cell density, long and total sub-basal nerve numbers	Decreased corneal basal epithelium, anterior and posterior keratocytes, endothelial cell density, meibomian gland density, and sub-basal nerve numbers in chronic smokers.	Systemic concentrations of cigarette toxic substances are not assessed in the blood, unclear whether ocular alterations due to systemic effects or direct damage from smoking.
UV Damage to the Anterior Ocular Surface	Grupcheva	Summer sun exposure leads to changes in the cornea, bulbar and palpebral conjunctiva	Prospective	400	Outpatient	2 (baseline, 1 year)	HRTII-RCM	No and area of cystic changes	Characteristic cystic lesions with dark centers and bright borders in only 25 eyes (6%) before and affecting 118 eyes (29.5%) after summer. The total area of the cysts after the summer increased fivefold.	Same population used as a control—may not have adequate time for washout of effects

CDCD: Corneal DC density; EC: epithelial cell; GCD: goblet cell density; GVHD: graft versus host disease; SNP: sub-basal nerve plexus.

**Table 7:** Studies on CL-Related Dry Eye.

Area of Study	Authors	Main Contribution to Literature	Design	Sample Size	Participants	No of IVCM	HRT	Main Outcomes	Main Findings	Limitations
Corneal Alterations of New Hybrid CL in Advanced Keratoconus	Dikmetas	Hybrid CL: no adverse effects on corneal endothelial cells in advanced keratoconus.	Retrospective study	32	Outpatient	2 (baseline, at 6 months)	IVCM; Confoscan4; Nidek	Corneal endothelial cell density	No significant reduction in epithelial cell density noted at the 6-month compared to baseline after wear	Retrospective design, limited sample size, no control group did not study nerve alterations
CL Wear on Corneal Epithelial DC Distribution, Density, and Morphology	Golebiowski	Density, distribution, and morphology of CEDC do not differ in established CL wearers	Investigator-masked cross-sectional observational pilot study	40	Outpatient	1	HRTII-RCM	Corneal epithelial DCs	Relatively lower density of corneal epithelial DCs in the central cornea of younger patients may allude to a more naive immune status in lens wearers	Small sample size
Microstructural Evaluation of Mucin Balls and Relations to Corneal Surface	Grupcheva	Mucin balls affect the corneal surface, including both epithelial disintegration as well as keratocyte “activation”.	Prospective case-control study	42	Outpatient	2 (baselined at 28 ± 2 days	HRT III-RCM	Appearance and size of the mucin balls Qualitative analysis of shape (round, elliptical, and irregular), reflectivity (bright, homogenous and dark, heterogonous).	Negative correlation between the size of balls and impact on basal epithelium morphology and “activation” of anterior stroma in adjacent areas	Small sample size
Silicone Hydrogel CL Wear and Corneal Sub-basal Nerve Plexus.	Kocabeyoglu	Sensory adaptation to CL wear is not mediated through sub-basal nerve or reduction of corneal tactile sensitivity in CL-naive users.	Prospective longitudinal study	40	Outpatient	2 (baseline, 6 months)	Confoscan 3.0 (Nidek, Vigonza, Italy)	Corneal sub-basal nerve densities mean total sub-basal nerve fiber length, mean total sub-basal nerve branch density, or the mean long nerve fiber density	No significant changes in outcomes at 6-month follow-up in CL users.	Small sample size
CL-Related Complications	Li, Weiwei	Complications related to CL wear-most common is dry eye, then SPK	Retrospective	141	Outpatient	1	Not specified	Visualizing Acanthoemoeba cysts, examining meibomian glands	No cysts found. Meibomian glands described	Mild or asymptomatic complications not observed Only one hospital
Changes in Tarsal Conjunctiva Associated With Ocular Symptoms and CL Wear	López-de la Rosa	Soft CL wear modifies papillae of epithelial-lamina propria junction into a more rounded shape; however, CL cessation appears to resolve this alteration.	Retrospective	92	Outpatient	1	HRT III-RCM	Papillae density, shortest diameter, longest diameter, area, circularity, lumen/wall brightness ratio, irregularity, reflectivity, inhomogeneous appearance of the wall, and inhomogeneous appearance of rete ridges	CL wearers, compared to previous wearers and non-wearers, showed higher circularity. Subjects with symptoms, compared to asymptomatic participants, showed higher circularity and lower irregularity	Retrospective nature of study, 2 different questionnaires used for CL wearers and non-CL-wearers, CL material type not controlled
Changes in Corneal Sub-basal Nerve Morphology and Sensitivity During OK	Lum, Edward	Alterations in corneal nerve morphology occur rapidly with OK and underpin functional sensitivity loss. Nerve fiber orientation provides an index for changes in corneal nerve morphology.	Prospective case-control study	39	Outpatient	3 (baseline, D30, D90)	HRT II RCM	NFD GNFO	In the central cornea, both NFD and corneal sensitivity decreased by Day 30, 90. Reduced NFD is associated with reduced corneal sensitivity. In the mid-peripheral cornea, GNFO rotated clockwise on Day 30, with further rotation on Day 90. Corneal sensitivity reduction plateaued by Day 30.	Difficulties in locating the same exact corneal location with IVCM at multiple visits for each subject, leading to a potential sampling error
Long-Term Impacts of OK on SNP and Corneal Sensitivity Responses and Their Reversibility	Nombela-Palomo	Long-term OK treatment led to reduced SNP nerve density, directly correlated with corneal tortuosity. After one month of treatment interruption, nerve density was still reduced.	Prospective case-control study	47	Outpatient	3 (baseline, one year, one month after removing lens)	Not specified	SNP	OK wearers: reductions in SNP density and no. of nerves in the central and mid-peripheral cornea Increased central objective tortuosity After lens removal for 1 month, baseline nerve density was not recovered. One year: Increased mid-peripheral Langerhans cell density, Increase in mid-peripheral nerve tortuosity.	Small sample size
Subclinical Inflammation of the Ocular Surface in Soft CL Wear	Saliman	Daily disposable CL produces minimal subclinical inflammatory response vs. no lens wear over 1 week.	Prospective, longitudinal, observational	20	Outpatient	6: 3 dispensing, 3 follow-up visits	HRT III/RCM	DC density DC morphology	All metrics increase in reusable lenses (A2 and AO), while only 3 of 6 IVCM parameters increase in daily disposable group.	Small sample size
Corneal Health during Three Months of Scleral Lens Wear	Tse V	Scleral lens wear for 3 months does not affect corneal epithelial barrier function, nerve fiber, and DC densities	Prospective, longitudinal, observational	27	Outpatient	3 (baseline, 1, 3 months)	HRT-RCM	Corneal epithelial permeability DC density Corneal Nerve Fiber Morphology Endothelial Cell Density	No differences between CL solutions. No changes after 1 and 3 months of CL use.	No comparison with patients who did not have a scleral lens or other lens types.
Impact of Lens Care Solutions and Daily Silicone Hydrogel CL Wear on Cornea Epithelium	Zhang XL	IVCM can detect epithelial cellular changes during CL wear	Prospective, investigator masked, cohort study	274	Outpatient	2 (baseline and at 5 months)	ConfoScan4 (Nidek)	Morphologic differences (hyper-reflectivity) in the superficial ECs and epithelial basal cell density	Hyper-reflective superficial ECs associated with PHMB preserved solution; decreased basal EC density associated with bacterial bioburden.	No washout period prior to study entry
Corneal DC and Sub-basal Nerve in Long-Term CL Wear	Liu Q	IVCM enabled direct observation of increased corneal DC and correlated with loss of SNP.	Prospective, observational	20	Outpatient	4 (1, 4, 12, and 24 weeks)	HRT II-RCM	Density of dendrites Area of dendrites No of dendrites, Total length of DC SNP densities in central and peripheral corneas	After wearing CLs, corneal DC were activated and increased, indicating ocular surface inflammation and decreased after week 4	Effect of gender not evaluated Small study

CL: contact lens; DC: dendritic cell; EC: epithelial cell; GNFO: global nerve fiber orientation NFD: nerve fiber density; Nerve fiber density (NFD); PHMB: polyhexamethylene biguanide; OK: orthokeratology; SNP: sub-basal nerve plexus.

## Data Availability

Not applicable.

## Supplementary Materials

The following are available online at https://www.mdpi.com/article/10.3390/jcm11092349/s1, Supplementary File S1: TFOS Lifestyle Workshop 2021—Standardised Electronic Searches. Table S1: Table to summarize the results of AMSTAR-2. Table S2: Risk of bias summary for individual studies (n = 4) in accordance with Rob2.

## References

[1] Cruzat A., Pavan-Langston D., Hamrah P. (2010). In Vivo Confocal Microscopy of Corneal Nerves: Analysis and Clinical Correlation. Semin. Ophthalmol..

[2] Dua H.S., Faraj L., Said D.G., Gray T., Lowe J. (2013). Human Corneal Anatomy Redefined. Ophthalmology.

[3] Bohn S., Sperlich K., Stahnke T., Schünemann M., Stolz H., Guthoff R.F., Stachs O. (2020). Multiwavelength confocal laser scanning microscopy of the cornea. Biomed. Opt. Express.

[4] Stachs O., Guthoff R.F., Aumann S. (2019). In Vivo Confocal Scanning Laser Microscopy. High Resolution Imaging in Microscopy and Ophthalmology.

[5] Khamar P., Nair A.P., Shetty R., Vaidya T., Subramani M., Ponnalagu M., Dhamodaran K., D’Souza S., Ghosh A., Pahuja N. (2019). Dysregulated Tear Fluid Nociception-Associated Factors, Corneal Dendritic Cell Density, and Vitamin D Levels in Evaporative Dry Eye. Investig. Opthalmol. Vis. Sci..

[6] Cruzat A., Qazi Y., Hamrah P. (2016). In Vivo Confocal Microscopy of Corneal Nerves in Health and Disease. Ocul. Surf..

[7] Schmidl D., Schlatter A., Chua J., Tan B., Garhöfer G., Schmetterer L. (2020). Novel Approaches for Imaging-Based Diagnosis of Ocular Surface Disease. Diagnostics.

[8] Binotti W.W., Bayraktutar B., Ozmen M.C., Cox S.M., Hamrah P. (2020). A Review of Imaging Biomarkers of the Ocular Surface. Eye Contact Lens Sci. Clin. Pract..

[9] Shea B.J., Reeves B.C., Wells G., Thuku M., Hamel C., Moran J., Moher D., Tugwell P., Welch V., Kristjansson E. (2017). AMSTAR 2: A critical appraisal tool for systematic reviews that include randomised or non-randomised studies of healthcare interventions, or both. BMJ.

[10] Sterne J.A.C., Savović J., Page M.J., Elbers R.G., Blencowe N.S., Boutron I., Cates C.J., Cheng H.Y., Corbett M.S., Eldridge S.M. (2019). RoB 2: A revised tool for assessing risk of bias in randomised trials. BMJ.

[11] Efron N., Al-Dossari M., Pritchard N. (2009). In Vivo Confocal Microscopy of the Palpebral Conjunctiva and Tarsal Plate. Optom. Vis. Sci..

[12] Chhadva P., Goldhardt R., Galor A. (2017). Meibomian Gland Disease: The Role of Gland Dysfunction in Dry Eye Disease. Ophthalmology.

[13] Zhao H., Chen J.-Y., Wang Y.-Q., Lin Z.-R., Wang S. (2016). In vivo Confocal Microscopy Evaluation of Meibomian Gland Dysfunction in Dry Eye Patients with Different Symptoms. Chin. Med. J..

[14] Qazi Y., Kheirkhah A., Blackie C., Cruzat A., Trinidad M., Williams C., Korb D.R., Hamrah P. (2015). In vivo detection of clinically non-apparent ocular surface inflammation in patients with meibomian gland dysfunction-associated refractory dry eye symptoms: A pilot study. Eye.

[15] Qazi Y., Kheirkhah A., Blackie C., Trinidad M., Williams C., Cruzat A., Korb D.R., Hamrah P. (2018). Clinically Relevant Immune-Cellular Metrics of Inflammation in Meibomian Gland Dysfunction. Investig. Opthalmol. Vis. Sci..

[16] Craig J.P., Nichols K.K., Akpek E.K., Caffery B., Dua H.S., Joo C.-K., Liu Z., Nelson J.D., Nichols J.J., Tsubota K. (2017). TFOS DEWS II Definition and Classification Report. Ocul. Surf..

[17] Azizi S., Uçak T., Yaşar I., Karakurt Y., Erdogan E., Salman I. (2017). Evaluation of the Corneal Layers in Meibomian-Gland-Dysfunction-Related Dry Eye by In Vivo Slit-Scanning Confocal Microscopy. Semin. Ophthalmol..

[18] Stern M.E., Schaumburg C.S., Pflugfelder S.C. (2013). Dry Eye as a Mucosal Autoimmune Disease. Int. Rev. Immunol..

[19] Al-Aqaba M.A., Dhillon V.K., Mohammed I., Said D.G., Dua H.S. (2019). Corneal nerves in health and disease. Prog. Retin. Eye Res..

[20] Liu Y.-C., Lin M.T.-Y., Mehta J.S. (2021). Analysis of corneal nerve plexus in corneal confocal microscopy images. Neural Regen. Res..

[21] Xu J., Chen P., Yu C., Liu Y., Hu S., Di G. (2021). In vivo Confocal Microscopic Evaluation of Corneal Dendritic Cell Density and Subbasal Nerve Parameters in Dry Eye Patients: A Systematic Review and Meta-analysis. Front. Med..

[22] Maruoka S., Inaba M., Ogata N. (2018). Activation of Dendritic Cells in Dry Eye Mouse Model. Investig. Opthalmol. Vis. Sci..

[23] Kallinikos P., Berhanu M., O’Donnell C., Boulton A.J.M., Efron N., Malik R. (2004). Corneal Nerve Tortuosity in Diabetic Patients with Neuropathy. Investig. Opthalmol. Vis. Sci..

[24] Tepelus T.C., Chiu G.B., Huang J., Huang P., Sadda S.R., Irvine J., Lee O.L. (2017). Correlation between corneal innervation and inflammation evaluated with confocal microscopy and symptomatology in patients with dry eye syndromes: A preliminary study. Graefe’s Arch. Clin. Exp. Ophthalmol..

[25] Jalbert I., Stapleton F., Papas E., Sweeney D., Coroneo M. (2003). In vivo confocal microscopy of the human cornea. Br. J. Ophthalmol..

[26] Liu Y., Chou Y., Dong X., Liu Z., Jiang X., Hao R., Li X. (2017). Corneal Subbasal Nerve Analysis Using In Vivo Confocal Microscopy in Patients with Dry Eye: Analysis and Clinical Correlations. Cornea.

[27] Ma B., Xie J., Yang T., Su P., Liu R., Sun T., Zhou Y., Wang H., Feng X., Ma S. (2021). Quantification of Increased Corneal Subbasal Nerve Tortuosity in Dry Eye Disease and Its Correlation with Clinical Parameters. Transl. Vis. Sci. Technol..

[28] Nguyen C.Q., Peck A.B. (2009). Unraveling the Pathophysiology of Sjogren Syndrome-Associated Dry Eye Disease. Ocul. Surf..

[29] Li F., Zhang Q., Ying X., He J., Jin Y., Xu H., Cheng Y., Zhao M. (2021). Corneal nerve structure in patients with primary Sjögren’s syndrome in China. BMC Ophthalmol..

[30] McNamara N.A., Ge S., Lee S.M., Enghauser A.M., Kuehl L., Chen F.Y.-T., Gallup M., McKown R.L. (2016). Reduced Levels of Tear Lacritin Are Associated With Corneal Neuropathy in Patients With the Ocular Component of Sjögren’s Syndrome. Investig. Opthalmol. Vis. Sci..

[31] Hillenaar T., van Cleynenbreugel H., Verjans G.M., Wubbels R.J., Remeijer L. (2012). Monitoring the Inflammatory Process in Herpetic Stromal Keratitis: The Role of In Vivo Confocal Microscopy. Ophthalmology.

[32] Lanza M., Iaccarino S., Varricchi G., D’Errico T., Carnevale U.A.G., Bifani M. (2016). Corneal confocal microscopy alterations in Sjögren’s syndrome dry eye. Acta Ophthalmol..

[33] Lee O.L., Tepelus T.C., Huang J., Irvine A.G., Irvine C., Chiu G.B., Sadda S.R. (2018). Evaluation of the corneal epithelium in non-Sjögren’s and Sjögren’s dry eyes: An in vivo confocal microscopy study using HRT III RCM. BMC Ophthalmol..

[34] Kawashima M., Sano K., Takechi S., Tsubota K. (2018). Impact of lifestyle intervention on dry eye disease in office workers: A randomized controlled trial. J. Occup. Health.

[35] Giannaccare G., Pellegrini M., Sebastiani S., Bernabei F., Roda M., Taroni L., Versura P., Campos E.C. (2019). Efficacy of Omega-3 Fatty Acid Supplementation for Treatment of Dry Eye Disease: A Meta-Analysis of Randomized Clinical Trials. Cornea.

[36] Leyva I.M., Molina-Leyva A., Bueno-Cavanillas A. (2017). Efficacy of nutritional supplementation with omega-3 and omega-6 fatty acids in dry eye syndrome: A systematic review of randomized clinical trials. Acta Ophthalmol..

[37] Levy O., Labbé A., Borderie V., Hamiche T., Dupas B., Laroche L., Baudouin C., Bouheraoua N. (2017). Increased corneal sub-basal nerve density in patients with Sjögren syndrome treated with topical cyclosporine A. Clin. Exp. Ophthalmol..

[38] Iaccheri B., Torroni G., Cagini C., Fiore T., Cerquaglia A., Lupidi M., Cillino S., Dua H.S. (2017). Corneal confocal scanning laser microscopy in patients with dry eye disease treated with topical cyclosporine. Eye.

[39] Giannaccare G., Buzzi M., Fresina M., Velati C., Versura P. (2017). Efficacy of 2-Month Treatment With Cord Blood Serum Eye Drops in Ocular Surface Disease: An In Vivo Confocal Microscopy Study. Cornea.

[40] Chen X., Graham J., Petropoulos I.N., Ponirakis G., Asghar O., Alam U., Marshall A., Ferdousi M., Azmi S., Efron N. (2018). Corneal Nerve Fractal Dimension: A Novel Corneal Nerve Metric for the Diagnosis of Diabetic Sensorimotor Polyneuropathy. Investig. Opthalmol. Vis. Sci..

[41] Villani E., Garoli E., Termine V., Pichi F., Ratiglia R., Nucci P. (2015). Corneal Confocal Microscopy in Dry Eye Treated with Corticosteroids. Optom. Vis. Sci..

[42] Chinnery H.R., Golborne C.N., Downie L.E. (2017). Omega-3 supplementation is neuroprotective to corneal nerves in dry eye disease: A pilot study. Ophthalmic Physiol. Opt..

[43] Mansoor H., Tan H.C., Lin M.T.-Y., Mehta J.S., Liu Y.-C. (2020). Diabetic Corneal Neuropathy. J. Clin. Med..

[44] Clerck E.E.B.D., Schouten J.S., Berendschot T.T., Kessels A.G.H., Nuijts R.M.M.A., Beckers H.J.M., Schram M., Stehouwer C.D.A., Webers C.A.B. (2015). New ophthalmologic imaging techniques for detection and monitoring of neurodegenerative changes in diabetes: A systematic review. Lancet Diabetes Endocrinol..

[45] Perkins B.A., Lovblom L.E., Bril V., Scarr D., Ostrovski I., Orszag A., Edwards K., Pritchard N., Russell A., Dehghani C. (2018). Corneal confocal microscopy for identification of diabetic sensorimotor polyneuropathy: A pooled multinational consortium study. Diabetologia.

[46] Liu Y.-C., So W., Wong N.Q., Tan H., Lin M.Y., Lee I.Y., Mehta J. (2022). Diabetic corneal neuropathy as a surrogate marker for diabetic peripheral neuropathy. Neural Regen. Res..

[47] Lagali N.S., Allgeier S., Guimarães P., Badian R.A., Ruggeri A., Köhler B., Utheim T.P., Peebo B., Peterson M., Dahlin L.B. (2017). Reduced Corneal Nerve Fiber Density in Type 2 Diabetes by Wide-Area Mosaic Analysis. Investig. Opthalmol. Vis. Sci..

[48] Utsunomiya T., Nagaoka T., Hanada K., Omae T., Yokota H., Abiko A., Haneda M., Yoshida A. (2015). Imaging of the Corneal Subbasal Whorl-like Nerve Plexus: More Accurate Depiction of the Extent of Corneal Nerve Damage in Patients With Diabetes. Investig. Opthalmol. Vis. Sci..

[49] Cozzini T., Piona C., Marchini G., Merz T., Brighenti T., Bonetto J., Marigliano M., Olivieri F., Maffeis C., Pedrotti E. (2021). In vivo confocal microscopy study of corneal nerve alterations in children and youths with Type 1 diabetes. Pediatr. Diabetes.

[50] Petropoulos I.N., Ferdousi M., Marshall A., Alam U., Ponirakis G., Azmi S., Fadavi H., Efron N., Tavakoli M., Malik R.A. (2015). The Inferior Whorl For Detecting Diabetic Peripheral Neuropathy Using Corneal Confocal Microscopy. Investig. Opthalmol. Vis. Sci..

[51] Li S., Liu D., Li L., Li Y., Li Q., An Z., Sun X., Tian H. (2016). Circulating Betatrophin in Patients with Type 2 Diabetes: A Meta-Analysis. J. Diabetes Res..

[52] Liang H., Randon M., Michee S., Tahiri R., Labbe A., Baudouin C. (2016). In vivo confocal microscopy evaluation of ocular and cutaneous alterations in patients with rosacea. Br. J. Ophthalmol..

[53] Gürdal C., Saraç Ö., Genç I., Kırımlıoğlu H., Takmaz T., Can I. (2011). Ocular Surface and Dry Eye in Graves’ Disease. Curr. Eye Res..

[54] Harrison A.R., Bothun E.D., Scheuer R.A., Lee M.S. (2009). Update on thyroid eye disease and management. Clin. Ophthalmol..

[55] Wu L.-Q., Mou P., Chen Z.-Y., Cheng J.-W., Le Q.-H., Cai J.-P., Wei R.-L. (2019). Altered Corneal Nerves in Chinese Thyroid-Associated Ophthalmopathy Patients Observed by In Vivo Confocal Microscopy. Med. Sci. Monit..

[56] Cheng S., Yu Y., Chen J., Ye L., Wang X., Jiang F. (2021). In vivo confocal microscopy assessment of meibomian glands microstructure in patients with Graves’ orbitopathy. BMC Ophthalmol..

[57] Yang S., Kim W., Kim H.S., Na K.-S. (2017). Epidemiologic Survey Committee of the Korean Ophthalmologic Society Association Between Migraine and Dry Eye Disease: A Nationwide Population-Based Study. Curr. Eye Res..

[58] Sarac O., Kosekahya P., Tasci Y.Y., Keklikoglu H.D., Deniz O., Erten Ş., Çağıl N. (2017). The Prevalence of Dry Eye and Sjögren Syndrome in Patients with Migraine. Ocul. Immunol. Inflamm..

[59] Situ P., Simpson T.L., Jones L.W., Fonn D. (2008). Conjunctival and Corneal Hyperesthesia in Subjects with Dryness Symptoms. Optom. Vis. Sci..

[60] Edvinsson J.C.A., Viganò A., Alekseeva A., Alieva E., Arruda R., De Luca C., D’Ettore N., Frattale I., Kurnukhina M., Macerola N. (2020). The fifth cranial nerve in headaches. J. Headache Pain.

[61] Shetty R., Deshmukh R., Shroff R., Dedhiya C., Jayadev C. (2017). Subbasal Nerve Plexus Changes in Chronic Migraine. Cornea.

[62] Tham Y.-C., Li X., Wong T.Y., Quigley H.A., Aung T., Cheng C.-Y. (2014). Global Prevalence of Glaucoma and Projections of Glaucoma Burden through 2040: A systematic review and meta-analysis. Ophthalmology.

[63] Fechtner R.D., Godfrey D.G., Budenz D., Stewart J.A., Stewart W.C., Jasek M.C. (2010). Prevalence of Ocular Surface Complaints in Patients With Glaucoma Using Topical Intraocular Pressure-Lowering Medications. Cornea.

[64] Servat J.J., Bernardino C.R. (2011). Effects of Common Topical Antiglaucoma Medications on the Ocular Surface, Eyelids and Periorbital Tissue. Drugs Aging.

[65] Kucukevcilioglu M., Bayer A., Uysal Y., Altinsoy H.I. (2013). Prostaglandin associated periorbitopathy in patients using bimatoprost, latanoprost and travoprost. Clin. Exp. Ophthalmol..

[66] Rolle T., Spinetta R., Nuzzi R. (2017). Long term safety and tolerability of Tafluprost 0.0015% vs Timolol 0.1% preservative-free in ocular hypertensive and in primary open-angle glaucoma patients: A cross sectional study. BMC Ophthalmol..

[67] Mastropasqua R., Fasanella V., Brescia L., Oddone F., Mariotti C., Di Staso S., Agnifili L. (2017). In Vivo Confocal Imaging of the Conjunctiva as a Predictive Tool for the Glaucoma Filtration Surgery Outcome. Investig. Opthalmol. Vis. Sci..

[68] Amar N., Labbé A., Hamard P., Dupas B., Baudouin C. (2008). Filtering Blebs and Aqueous Pathway: An Immunocytological and In Vivo Confocal Microscopy Study. Ophthalmology.

[69] Agrawal A., Agrawal S., Gupta S. (2017). Role of Dendritic Cells in Inflammation and Loss of Tolerance in the Elderly. Front. Immunol..

[70] Sacchi M., Agnifili L., Brescia L., Oddone F., Villani E., Nucci P., Mastropasqua L. (2020). Structural imaging of conjunctival filtering blebs in XEN gel implantation and trabeculectomy: A confocal and anterior segment optical coherence tomography study. Graefe’s Arch. Clin. Exp. Ophthalmol..

[71] Herretes S., Ross D.B., Duffort S., Barreras H., Yaohong T., Saeed A.M., Murillo J.C., Komanduri K.V., Levy R.B., Perez V.L. (2015). Recruitment of Donor T Cells to the Eyes During Ocular GVHD in Recipients of MHC-Matched Allogeneic Hematopoietic Stem Cell Transplants. Investig. Opthalmol. Vis. Sci..

[72] Tepelus T.C., Chiu G.B., Maram J., Huang J., Chopra V., Sadda S.R., Lee O.L. (2017). Corneal features in ocular graft-versus-host disease by in vivo confocal microscopy. Graefe’s Arch. Clin. Exp. Ophthalmol..

[73] Niederer R., Perumal D., Sherwin T., McGhee C.N.J. (2007). Corneal Innervation and Cellular Changes after Corneal Transplantation: An In Vivo Confocal Microscopy Study. Investig. Opthalmol. Vis. Sci..

[74] Patel S.V., Erie J.C., McLaren J.W., Bourne W.M. (2007). Keratocyte and subbasal nerve density after penetrating keratoplasty. Trans. Am. Ophthalmol. Soc..

[75] Al-Aqaba M.A., Otri A.M., Fares U., Miri A., Dua H.S. (2012). Organization of the Regenerated Nerves in Human Corneal Grafts. Am. J. Ophthalmol..

[76] Ogawa Y. (2018). Sjögren’s Syndrome, Non-Sjögren’s Syndrome, and Graft-Versus-Host Disease Related Dry Eye. Investig. Opthalmol. Vis. Sci..

[77] Dikmetas O., Kocabeyoglu S., Mocan M.C. (2021). The Association between Meibomian Gland Atrophy and Corneal Subbasal Nerve Loss in Patients with Chronic Ocular Graft-versus-host Disease. Curr. Eye Res..

[78] Stapleton F., Keay L., Jalbert I., Cole N. (2007). The Epidemiology of Contact Lens Related Infiltrates. Optom. Vis. Sci..

[79] Poggio E.C., Glynn R.J., Schein O.D., Seddon J.M., Shannon M.J., Scardino V.A., Kenyon K.R. (1989). The Incidence of Ulcerative Keratitis among Users of Daily-Wear and Extended-Wear Soft Contact Lenses. N. Engl. J. Med..

[80] Chalmers R.L., Keay L., McNally J., Kern J. (2012). Multicenter Case-Control Study of the Role of Lens Materials and Care Products on the Development of Corneal Infiltrates. Optom. Vis. Sci..

[81] Golebiowski B., Chao C., Bui K.A., Lam W.Y.W., Richdale K., Stapleton F. (2019). Effect of age and contact lens wear on corneal epithelial dendritic cell distribution, density, and morphology. Contact Lens Anterior Eye.

[82] Saliman N.H., Morgan P., MacDonald A.S., Maldonado-Codina C. (2019). Subclinical Inflammation of the Ocular Surface in Soft Contact Lens Wear. Cornea.

[83] Steele K.R., Szczotka-Flynn L. (2017). Epidemiology of contact lens-induced infiltrates: An updated review. Clin. Exp. Optom..

[84] Lum E., Golebiowski B., Swarbrick H.A. (2017). Reduced Corneal Sensitivity and Sub-Basal Nerve Density in Long-Term Orthokeratology Lens Wear. Eye Contact Lens Sci. Clin. Pract..

[85] Nombela-Palomo M., Felipe-Márquez G., Teus M., Hernández-Verdejo J.L., Bona A.N. (2018). Long-Term Impacts of Orthokeratology Treatment on Sub-Basal Nerve Plexus and Corneal Sensitivity Responses and Their Reversibility. Eye Contact Lens Sci. Clin. Pract..

[86] Turuwhenua J.T., Patel D., McGhee C.N. (2012). Fully Automated Montaging of Laser Scanning In Vivo Confocal Microscopy Images of the Human Corneal Subbasal Nerve Plexus. Investig. Opthalmol. Vis. Sci..

[87] Badian R.A., Allgeier S., Scarpa F., Andréasson M., Bartschat A., Mikut R., Colonna A., Bellisario M., Utheim T.P., Köhler B. (2021). Wide-field mosaics of the corneal subbasal nerve plexus in Parkinson’s disease using in vivo confocal microscopy. Sci. Data.

[88] Bohr A., Memarzadeh K. (2020). The rise of artificial intelligence in healthcare applications. Artificial Intelligence in Healthcare.

