# Supplementary material for: In Vivo Confocal Microscopy in Different Types of Dry Eye and Meibomian Gland Dysfunction
Source: J Clin Med. 2022 Apr 22;11(9):2349. doi: 10.3390/jcm11092349 (PMC9099706; doi:10.3390/jcm11092349)
Supplement: Supplementary file 1 [file jcm-11-02349-s001.zip › Supplementary File S1.pdf]

<u>**TFOS Lifestyle Workshop 2021 – Standardised Electronic Searches**</u>

PubMed – “Ocular Surface” and “Ocular Surface Disease” Search Strategy

1. ("Diagnostic Techniques, Ophthalmological"[Mesh] OR "Eye Diseases"[Mesh] OR "Eye Injuries"[Mesh] OR "Eye"[Mesh] OR "Ocular Physiological Phenomena"[Mesh] OR "Optical Phenomena"[Mesh]) - **3184 results**
2. (vision[all] OR sight*[all] OR ocular[all] OR occular[all] OR limbus[all] OR limbal[all] OR orbit*[all] OR blink*[all] OR brow[all] OR canthus[all] OR canthal[all] OR conjunctiv*[all] OR cornea*[all] OR corneo*[all] OR eyel*[all] OR “eye lid*”[all] OR “eye lash*”[all] OR eyeb*[all] OR “eye brow*”[all] OR episcler*[all] OR lacrima*[all] OR goblet cell*[all] OR “lid wiper”[all] OR meibomi*[all] OR orbicularis[all] OR “Palisades of Vogt”[all] OR subconjunctiva*[all] OR tear[all] OR tears[all]) - **4158 results**
3. (Blepharitis[all] OR Blepharospasm[all] OR blindness[all] OR cataract*[all] OR chalazi*[all] OR chemosis[all] OR chemotic[all] OR CLADE[all] CLIDE[all] OR CLAPC[all] OR dacryo*[all] OR Demod*[all] OR distichiasis[all] OR DLK[all] OR “dry eye*” OR Ectropi*[all] OR Entropi*[all] OR Epiphora[all] OR ecchymosis[all] OR Exophthalm*[all] OR “eye pruritus”[all] OR “eye strain”[all] OR “globe rupture*”[all] OR Hordeol*[all] OR Keratitis[all] OR Keratopath*[all] OR “lid parallel”[all] OR “lid-parallel”[all] OR Limbitis[all] OR LIPCOF[all] OR Keratoconjunctivit*[all] OR Keratocon*[all] OR keratoplasty[all] OR lagophthalmos[all] OR madarosis[all] OR mascaroma[all] OR Oculopath*[all] OR Periorbital Fat Herniation[all] OR (Periocular[all] AND carcinoma*[all]) OR Photalgia[all] OR Photophobia[all] OR Photopsia[all] OR pinguec*[all] OR poliosis[all] OR “preseptal cellulitis”[all] OR “orbital cellulitis”[all] OR postkeratoplasty[all] OR post-keratoplasty[all] OR pteryg*[all] OR ptosis[all] OR ptotic[all] OR Scleral Disease*[all] OR Scleritis[all] OR Sicca*[all] OR Symblepharon[all] OR trichiasis[all] OR Xanthelasma[all] OR Xerophthalmi*[all] OR onchocercias*[all] OR trachoma*[all])- **1346 results**
4. #1 OR #2 OR #3

Ovid Medline

1. exp Diagnostic Techniques, Ophthalmological/
2. exp Eye Diseases/
3. exp Eye Injuries/
4. exp Eye/
5. exp Ocular Physiological Phenomena/
6. exp Optical Phenomena/
7. 1 OR 2 OR 3 OR 4 OR 5 OR 6
8. (vision or sight* or ocular or occular or limbus or limbal or orbit* or blink* or brow or canthus or canthal or conjunctiv* or cornea* or corneo* or eyel* or eye lid* or eye lash* or eyeb* or eye brow* or episcler* or lacrima* or goblet cell* or lid wiper or meibomi* or orbicularis or Palisades of Vogt or subconjunctiva* or tear or tears).af.
9. (blepharitis or blepharospasm or blindness or cataract* or chalazi* or chemosis or chemotic or CLADE or CLIDE or CLAPC or dacryo* or Demod* or distichiasis or DLK or dry eye* or ectropi* or entropi* or epiphora or ecchymosis or exophthalm* or eye

pruritis or eye strain or globe rupture* or hordeola* or keratitis or keratopath* or lid parallel or lid-parallel or limbitis or LIPCOF or keratoconjunctivit* or keratocon* or keratoplasty or lagophthalmos or madarosis or mascaroma or oculopathy* or periorbital fat herniation or (periocular AND carcinoma*) or photalgia or photophobia or photopsia or pinguec* or poliosis or preseptal cellulitis or orbital cellulitis or postkeratoplasty or post-keratoplasty or pteryg* or ptosis or ptotic or scleral disease* or scleritis or sicca* or symblepharon or trichiasis or xanthelasma or xerophthalmi or onchocercias* or trachoma*).af.

10. 7 OR 8 OR 9

Ovid Embase

1 exp ophthalmology/
2 exp eye/
3 exp eye disease/
4 exp eye injury/
5 diagnostic procedure/ and eye examination/
6 exp visual system function/
7 exp light related phenomena/
8 (vision or sight* or ocular or occular or limbus or limbal or orbit* or blink* or brow or canthus or canthal or conjunctiv* or cornea* or corneo* or eyel* or eye-lid* or eye-lash* or eyeb* or eye-brow* or episcler* or lacrima* or goblet-cell* or lid-wiper or meibomi* or orbicularis or Palisades-of-Vogt or subconjunctiva* or tear or tears).af.
9 (blepharitis or blepharospasm or blindness or cataract* or chalazi* or chemosis or chemotic or CLADE or CLIDE or CLAPC or dacryo* or Demod* or distichiasis or DLK or dry eye* or ectropi* or entropi* or epiphora or ecchymosis or exophthalm* or eye-pruritis or eye-strain or globe-rupture* or hordeola* or keratitis or keratopath* or lid-parallel or limbitis or LIPCOF or keratoconjunctivit* or keratocon* or keratoplasty or lagophthalmos or madarosis or mascaroma or oculopathy* or periorbital-fat-herniation or (periocular and carcinoma*) or photalgia or photophobia or photopsia or pinguec* or poliosis or preseptal-cellulitis or orbital-cellulitis or postkeratoplasty or post-keratoplasty or pteryg* or ptosis or ptotic or scleral-disease* or scleritis or sicca* or symblepharon or trichiasis or xanthelasma or xerophthalmi or onchocercias* or trachoma*).af.
10 1 or 2 or 3 or 4 or 5 or 6 or 7 or 8 or 9
