# Supplementary material for: In Vivo Confocal Microscopy in Different Types of Dry Eye and Meibomian Gland Dysfunction
Source: J Clin Med. 2022 Apr 22;11(9):2349. doi: 10.3390/jcm11092349 (PMC9099706; doi:10.3390/jcm11092349)
Supplement: Supplementary file 1 [file jcm-11-02349-s001.zip › Supplementary Tables S1 and S2.pdf]

Supplementary Table S1: Table to summarize the results of AMSTAR-2.

Questions	Response	More Information
Q1	Yes	Research questions include population, intervention, comparator and outcome
Q2	Yes	Review methods were established prior to conduct of the review (review question, search strategy, inclusion\exclusion, risk of bias
Q3	Yes	Inclusion of randomized and non-randomized studies (because there are very few randomized studies)
Q4	Partial yes	Searched only 1 database instead of 2, provided key word and search strategy
Q5	Yes	Two reviewers selected a sample of eligible studies and achieved good agreement, with the remainder selected by one reviewer
Q6	Yes	Two reviewers extracted data from a sample of eligible studies and achieved good agreement, with the remainder extracted by one reviewer
Q7	No	List of excluded articles available on request but too lengthy to be included in review article
Q8	Yes	Described population, intervention, comparators, outcomes and research designs
Q9	Yes	Mentioned whether lack of randomization or lack of blinding in studies
Q10	No	Did not include source of funding in review
Q11	No	No meta-analysis, too heterogenous
Q12	No	No meta-analysis
Q13	Yes	Included discussion of bias in intervention studies as well as cross sectional and observational studies
Q14	Yes	Mentioned the effects of heterogeneity as most studies are not interventional and not evaluating IVCM per se
Q15	No	No meta-analysis conducted
Q16	Yes	Authors reported no competing interest and sources of funding

Supplementary Table S2: Risk of bias summary for individual studies (n= 4) in accordance with Rob2.

Study	Yang	Postorino	Chinnery	Giannaccare
Outcome being assessed for ROB	10 outcomes listed, no primary outcome	6 outcomes listed, no primary outcome	Change in corneal sub-basal nerve plexus parameters	7 outcomes listed, no primary outcome
Effect	Assignment to intervention	Assignment to intervention	Assignment to intervention	Assignment to intervention
Source	Journal article	Journal article	Journal article	Journal article
Randomization	Yes	Yes	Yes	Yes
Deviation from intended intervention (includes masking)	Participant and investigator masked	Participant not masked *	Participant and investigator masked	Participant and investigator masked
Missing outcome data	Not specified	Not specified	Not specified	Not specified
Measurement bias	Investigator masked	Investigator masked	Investigator masked	Investigator masked
Selection of reported result	Multiple outcome measurements *	Multiple outcome measurements *	No clear bias in reporting	Multiple outcome measurements *
Overall bias	Direction of bias cannot be predicted	Direction of bias cannot be predicted	Direction of bias cannot be predicted	Direction of bias cannot be predicted
